# NiO/CuO@Graphene oxide-modified electrode for sensitive detection of an antidiabetic drug

**DOI:** 10.1038/s41598-025-32562-1

**Published:** 2026-01-06

**Authors:** Essam N. Labeeb, Mahmoud A. Hefnawy, Shymaa S. Medany, Eman Yossri Frag

**Affiliations:** Chemistry Department, Faculty of Science, Cairo University, Giza, 12613 Egypt

**Keywords:** Chemically modified electrode, Differential pulse voltammetry, Electrochemical sensor, Sitagliptin phosphate monohydrate, Chemistry, Materials science, Nanoscience and technology

## Abstract

A new, simple, and cost-effective electrochemical sensor was developed for the determination of Sitagliptin phosphate monohydrate (SP) in powder, pharmaceutical tablet form and biological fluids as spiked plasma. Electrochemical sensor used a chemically modified electrode (CME) based on a glassy carbon electrode (GCE) modified with graphene oxide (GO) and nickel/copper oxide nanoparticles (NiO/CuO NPs). The crystalline nature of GO/ (NiO/CuO NPs), the particle size of (NiO/CuO NPs) and the surface topography of the prepared surface were investigated using surface analysis techniques such as X-ray diffraction (XRD), transmission electron microscopy (TEM) and scanning electron microscopy (SEM) combined with energy dispersive X-ray analysis (EDXA) and elemental mapping. Surface characterization revealed that NiO/CuO NPs had an average size of 27.5 ± 2 nm and were uniformly distributed homogenously on the surface of GO. This distribution increased the effective surface area and enhanced electron transfer, confirming the structural and electrochemical advantages of the modified electrode material. The sensor efficiency was validated by different electrochemical techniques such as differential pulse voltammetry (DPV), cyclic voltammetry (CV), and electrical impedance (EIS). The Voltammetric response of SP powder form toward sensor was observed at − 0.1 V in DPV and − 0.055 V in CV, using 0.1 M phosphate-buffered solution (PBS, pH 7.4) as the electrolyte. The linear concentration range for SP in powder form was 0.05–1.071 mM, with a limit of detection (LOD) of 0.0223 mM and a limit of quantification (LOQ) of 0.0677 mM while, for powder in spiked plasma linear range was 0.0295–0.2715 mM, with LOD of 0.0061 mM and LOQ of 0.0185 mM. The diffusion coefficient (D) of 0.614 mM SP in PBS (pH 7.4, 0.1 mol/L) was evaluated using CV at various scan rates and found to be 2.41 × 10⁻⁶ cm²/s, based on the Randles–Sevcik equation. The method was validated by detecting SP in both powder and tablet forms using an Agilent 1200 HPLC system equipped with a UV detector at 266 nm. Statistical calculations such as recovery and precision were carried out to validate the accuracy and reliability of the method. The recovery values for both the electrochemical and HPLC-UV methods ranged from 99% to 101%, while the repeatability and reproducibility of the electrochemical method showed RSD values below 1.5%. The sensor demonstrated good sensitivity and selectivity toward SP, even in the presence of potential interferents such as glucose, ascorbic acid, and metal ions.

**Supplementary Information:**

The online version contains supplementary material available at 10.1038/s41598-025-32562-1.

## Introduction

Diabetes mellitus is a chronic disease that affects humans and is generally characterized by high blood glucose levels, either due to a lack of insulin or the body’s resistance to insulin. Glucose is the primary source of energy in the body, as all nutrients are eventually converted into glucose through digestion and metabolism^1^. Diabetes can affect people of all ages, including children, teenagers, women, and men, but it is especially common in individuals over 65 years old. Type 2 diabetes mellitus (T2D) occurs when the body produces low insulin, but the cells do not respond to it properly—a condition known as insulin resistance. The majority of diabetes cases worldwide are T2D, accounting for up to 95%^2^. The prevalence of T2D is attributed to various factors, including socio-economic, demographic, environmental, and genetic influences. Common causes include genetic predisposition, unhealthy lifestyles, physical inactivity, obesity, and high-carbohydrate diets. Symptoms of T2D include fatigue, excessive thirst, frequent urination, muscle weakness, and weight loss^3^. Currently, there is no definitive cure for T2D, but various treatments are available to manage the condition.

Many pharmaceutical drugs with different mechanisms of action provide effective control of blood glucose levels. SP is an oral antidiabetic drug approved by the US FDA in 2006. It has a molecular weight of approximately 523.32 g/mol and is used for patients with T2D; it is not suitable for those with type 1 diabetes mellitus (T1D). The mechanism of action of SP involves the inhibition of the enzyme dipeptidyl peptidase-4 (DPP-4). This enzyme normally degrades incretin hormones, which are responsible for increasing insulin secretion and decreasing glucagon release from pancreatic cells. By inhibiting DPP-4, SP helps regulate blood glucose levels and control hyperglycemia^4^. Like any drug taken, SP may cause common side effects, such as: headaches, fatigue, and upper respiratory tract infections but generally, it does not lead to hypoglycemia unless combined with other hypoglycemic agent may lead to hypoglycemia. SP may lead to pancreatitis with symptoms as continual vomiting, nausea and abdominal pain.

Many analytical methods have been employed for drug analysis at micro and trace concentrations, including spectrophotometric, chromatographic, electrophoretic, and electrochemical techniques^5^. According to the literature, several methods have been reported for the determination of sitagliptin (STG) or its phosphate form SP in pharmaceutical formulations and biological fluids, either alone or in combination with other antidiabetic drugs such as metaformin, simvastatin, and linagliptin. Some of previous methods were collected in Table S1. These reported and validated methods, including spectrophotometric as (UV, UV-visible, UV-FTIR)^6,7^, spectrofluorimetric methods^8^, chromatographic as (LC-MS/MS^9,10^, HPLC^11^, HPTLC^12–14^, UHPLC^15^, RP-HPLC^16^, and electrophoretic as (CZE^17^. Only a few electrochemical methods have been reported^18–21^.

Nowadays, electrochemical techniques specially Voltammetric techniques have attracted significant attention of scientists in the field of detection due to their numerous advantages, including high accuracy, sensitivity, selectivity, low cost, simple sample preparation, and rapid performance^22–26^. The most widely used electrochemical techniques for drug detection include potentiometry, amperometry, voltammetry, conductometry, and electrical impedance spectroscopy^27–32^. Voltammetric techniques mainly depend on working electrodes, where drug undergoes electron transfer reactions on surface of modified working electrodes so working electrode directly control sensitivity, selectivity, and sensor performance^33,34^. Electrochemical sensors are among the most extensively used in analytical chemistry due to their enhanced advantages, such as low detection limits, high sensitivity, and rapid response. In these systems, the generated electrical signal (typically current) is proportional to the analyte concentration^35–40^.

GCE is one of the most widely used working electrodes for CME in electrochemical techniques, as it offers several advantages over other electrodes such as carbon paste electrodes (CPE), screen-printed electrodes, and sonogel electrodes^41–45^. These advantages include excellent electrocatalytic properties, high electrical conductivity, low cost, a wide potential window, inertness, and chemical stability^46–48^. GO is a nanomaterial with a layered carbon structure and oxygen-containing functional groups (= O, –OH, –O–, –COOH)^49–51^. These structural features collectively contribute to its remarkable properties, including a high surface area, exceptional electron transfer properties, and enhanced conductivity, selectivity, and sensitivity^52–54^. Finally, NiO/CuO NPs are considered promising materials for electrocatalysis and sensing applications. Generally, NiO and CuO enhance catalytic activity due to their multiple oxidation states (Ni³⁺/Ni²⁺ and Cu²⁺/Cu⁺) which facilitate efficient charge transfer and accelerate reaction kinetics, thereby significantly lowering the overpotential required for the oxidation of sitagliptin phosphate monohydrate and increasing the current response. The development of CME is critical for the sensitive and selective detection of SP, offering a significant advancement over traditional methods.

This method aims to design a new electrochemical sensor that is accurate, sensitive, selective, and simple for the electrochemical determination of an antidiabetic drug SP, using CME based on GCE/GO/NiO/CuO NPs. The sensor is intended for applications like therapeutic drug monitoring and point-of-care (PoC) analysis in addition to use in pharmaceutical formulations or biological fluids such as spiked plasma. The novelty of purposed GCE/GO/NiO/CuO NPS modified electrode lies in its unique structure. This composite material enhances the electrode’s ability to facilitate the redox reaction of SP, leading to higher sensitivity, a lower detection limit, and improved selectivity. Sensor offers significant advantages in portability, cost-effectiveness, and ease of use, which are critical for on-site diagnostics.

## Experimental

### Chemicals and materials

The chemicals and materials used in this study were:

graphite, sulfuric acid (H₂SO₄) 98%, phosphoric acid (H₃PO₄) 85%, potassium permanganate (KMnO₄), hydrogen peroxide (H₂O₂) 30%, hydrochloric acid (HCl) 37%, ethanol (CH₃CH₂OH), nickel nitrate hexahydrate [Ni(NO₃)₂·6 H₂O], copper nitrate trihydrate [Cu(NO₃)₂·3 H₂O], ammonium hydroxide (NH₄OH), dipotassium hydrogen phosphate (K₂HPO₄), potassium dihydrogen phosphate (KH₂PO₄), sodium hydroxide (NaOH), glucose (C₆H₁₂O₆), ascorbic acid (C₆H₈O₆), sodium sulfate anhydrous (Na₂SO₄), copper sulfate pentahydrate (CuSO₄·5 H₂O), potassium chloride (KCl), SP powder (research grade), and JANUVIA^®^ tablets (50 mg, Merck Sharp & Dohme). All solid chemicals were purchased from Sigma-Aldrich with high purity (≥ 99%, analytical grade) and used without further purification. Double distilled water was employed to prepare the solutions and washing of the electrodes.

### Synthesis of graphene oxide (GO)

According to the modified Hummers’ method, GO can be prepared by the oxidation of graphite flakes (see Figure S1). Initially, graphite powder is added to a mixture of H₂SO₄ and H₃PO₄ in a 9:1 ratio under continuous stirring^55^. KMnO₄ is then slowly added to the solution in an ice bath while maintaining stirring. To terminate the oxidation process, H₂O₂ 30% is gradually added along with deionized water, still under ice bath conditions and constant stirring. The resulting mixture is then washed several times with HCl 10% and filtered to remove residual metal ions and salts. Finally, the washed graphene oxide is dried in an oven to obtain GO powder^56–58^.

### Preparation of nickel oxide/copper oxide nanoparticles (NiO/CuO NPs)

NiO/CuO NPs were synthesized using a hydrothermal method (see Figure S2). Aqueous solutions of Ni(NO₃)₂·6 H₂O and Cu(NO₃)₂·3 H₂O were prepared in 100 mL of distilled water at a molar ratio of 7:3. The pH of the solution was then adjusted to 9 using NH₄OH. The resulting precipitate was transferred to a sealed Teflon-lined autoclave and heated at 160 °C for 12 h. After cooling, the product was collected and washed several times with distilled water and ethanol. Finally, the obtained material was calcined in a furnace at 500 °C for 3 h to ensure the formation of NiO/CuO NPs^59,60^.

### Designing of chemically modified electrode (CME)

CME was prepared using GCE with a surface area of 0.707 cm² and a diameter of 3 mm. The GCE was first polished with a 2 μm alumina–water slurry to remove any surface scratches, then thoroughly cleaned with ethanol followed by double-distilled water. GO powder (2 mg) was mixed with NiO/CuO NPs (4 mg) in 1 mL of ethanol and subjected to ultrasonic treatment for 1 h to ensure uniform dispersion. The resulting electrocatalyst suspension was then drop-casted onto the surface of the cleaned GCE using a micropipette and allowed to dry at room temperature^61^.

### Preparation of real samples

A Januvia tablet STG 50 mg was ground and dissolved in 10 mL of PBS, pH 7.4, 0.1 mol/L using sonication. This 50 mg tablet is equivalent to 64.25 mg of SP, resulting in a final stock solution of SP with concentration (12.28 mM). This stock was then used to prepare a series of diluted solutions with the following concentrations (in mM):8.99, 6.42, 3.85 and 1.28. Under optimized DPV conditions, the electrochemical response of SP toward the modified electrode was observed and voltammograms obtained.

A blood sample was voluntarily donated by the first author. The blood was collected into a tube containing EDTA as anticoagulant. The sample was then centrifuged using a Digicen 25 centrifuge at 3000 rpm for 10 min to separate the plasma from blood cells. A 50 µL aliquot of plasma was diluted with 10 mL of PBS, pH 7.4, 0.1 mol/L at a 1:200 ratio for subsequent electrochemical analysis. Following the dilution, 100 µL of SP was added in successive aliquots to the spiked plasma solution. The electrochemical response was measured using modified electrode of GCE/GO/ NiO/CuO NPs, and voltammograms were obtained after each addition.

### Surface analysis and characterization

The surface of the GO/NiO/CuO NPs composite was examined using an X’Pert powder diffractometer for XRD analysis to identify the crystal structure of the prepared material^60^. TEM was used to determine the particle size of the (NiO/CuO NPs) distributed into the GO sheets^62^. The TEM analysis was conducted using a JEOL 1010 instrument, and the particle size was measured using ImageJ software. A histogram representing the particle size distribution was generated using Origin 9.0. SEM was employed to examine a film of the GO/NiO/CuO NPs composite deposited on a zinc substrate and coated with gold to prevent surface charging. The gold coating was applied using a DESK SPUTTER COATER (DSR1–VAC COAT). SEM analysis was carried out using a FEI Quanta FEG 250 instrument, which was equipped with EDXA and elemental mapping capabilities to determine the elemental composition of the material.

### Electrochemical measurements

A three-electrode electrochemical cell was employed, in which an Ag/AgCl (saturated KCl) electrode served as the reference electrode. The working electrode was a CME fabricated as GCE/GO/NiO/CuO NPs, while a platinum wire was used as the counter electrode. The electrolyte used for the measurements was PBS 0.1 M, with the pH adjusted to 7.4 using either H₃PO₄ or NaOH. Electrochemical techniques such as CV and DPV were performed using a Voltalab PGZ128N galvanostat/potentiostat (Radiometer Analytical, USA) with an error margin of ± 5 mV, and a potential range from 100 to 800 mV. EIS measurements were also carried out using the same instrument, with a modified AC voltage amplitude of 10 mV and a frequency range from 1 × 10⁴ Hz to 0.1 Hz. The resulting Nyquist plots and equivalent circuits were fitted using Nova software (version 2.1, Metrohm Autolab, Utrecht, Netherlands).

## Results and discussion

### Characterization and analysis

**Fig. 1:**
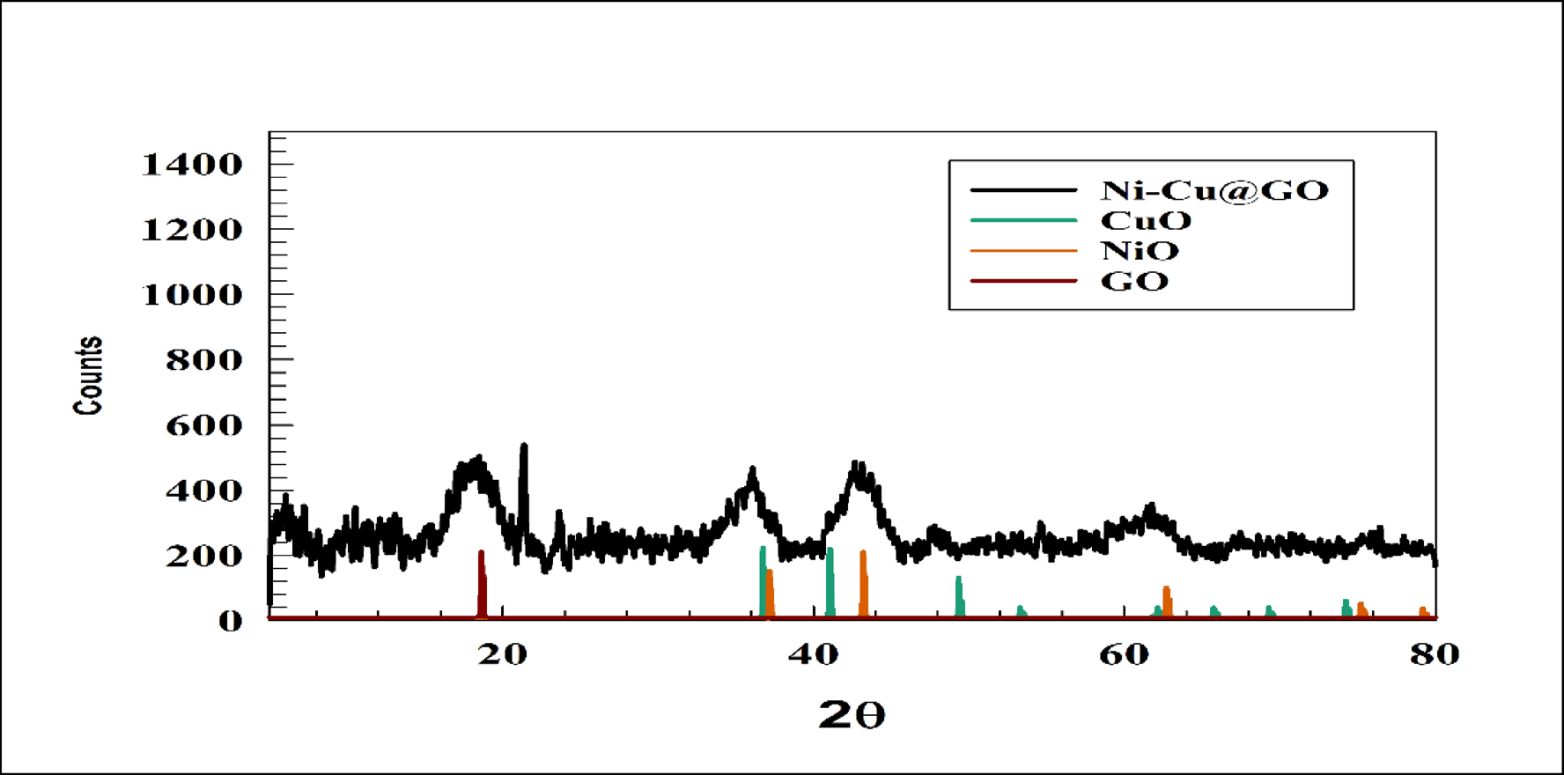
XRD pattern for GO/NiO/CuO NPs.

XRD is a valuable technique used to reveal crystalline properties such as texture, defects, phases, and crystal size^63,64^. This crystallinity is essential for predictable and stable electrochemical behavior. A well-defined crystal structure ensures a consistent and efficient pathway for ion and electron transport within the material. Figure 1 shows chart of XRD for the prepared surface. According to previous literature, there are no definitive XRD patterns or JCPDS card numbers for GO, as it may be non-crystalline, amorphous, or mildly crystalline in nature. A broad Hump was observed in the XRD pattern at 2theta ~ 20, attributed to the amorphous part for GO. Furthermore, the peak sharp peak appeared at 2thetal ~ 22, corresponding to (002). This peak depends on the degree of oxidation of graphite and interlayer spacing between sheets of oxidized graphite. The XRD pattern shows peaks at 2θ = 37.08°, 43.12°, 62.65°, 74.80°, and 78.90°, corresponding to the (111), (200), (220), (311), and (222) planes, respectively, of the cubic NiO crystal structure^65,66^. However, the peak observed for NiO matched with the reference card JCPDS 65-2901. Additionally, for the monoclinic phase of CuO, the main XRD peaks appeared at 2θ = 36.7°, 38.86°, 48.62°, 53.34°, 61.90°, 65.80°, 68.90°, and 74.30°, corresponding to the (002), (111), (202), (020), (113), (311), (220), and (004) planes, respectively. The peak for CuO matched with reference card number JCPDS 48-1548. However, the peak resolution of CuO was observed to be lower than expected due to the lower percentage of Cu compared to graphene oxide and nickel oxide. An overlap between NiO and CuO peaks was observed, which is attributed to the similarity of certain interplanar spacings (d-values), even though NiO has a cubic structure and CuO has a monoclinic structure.

**Fig. 2:**
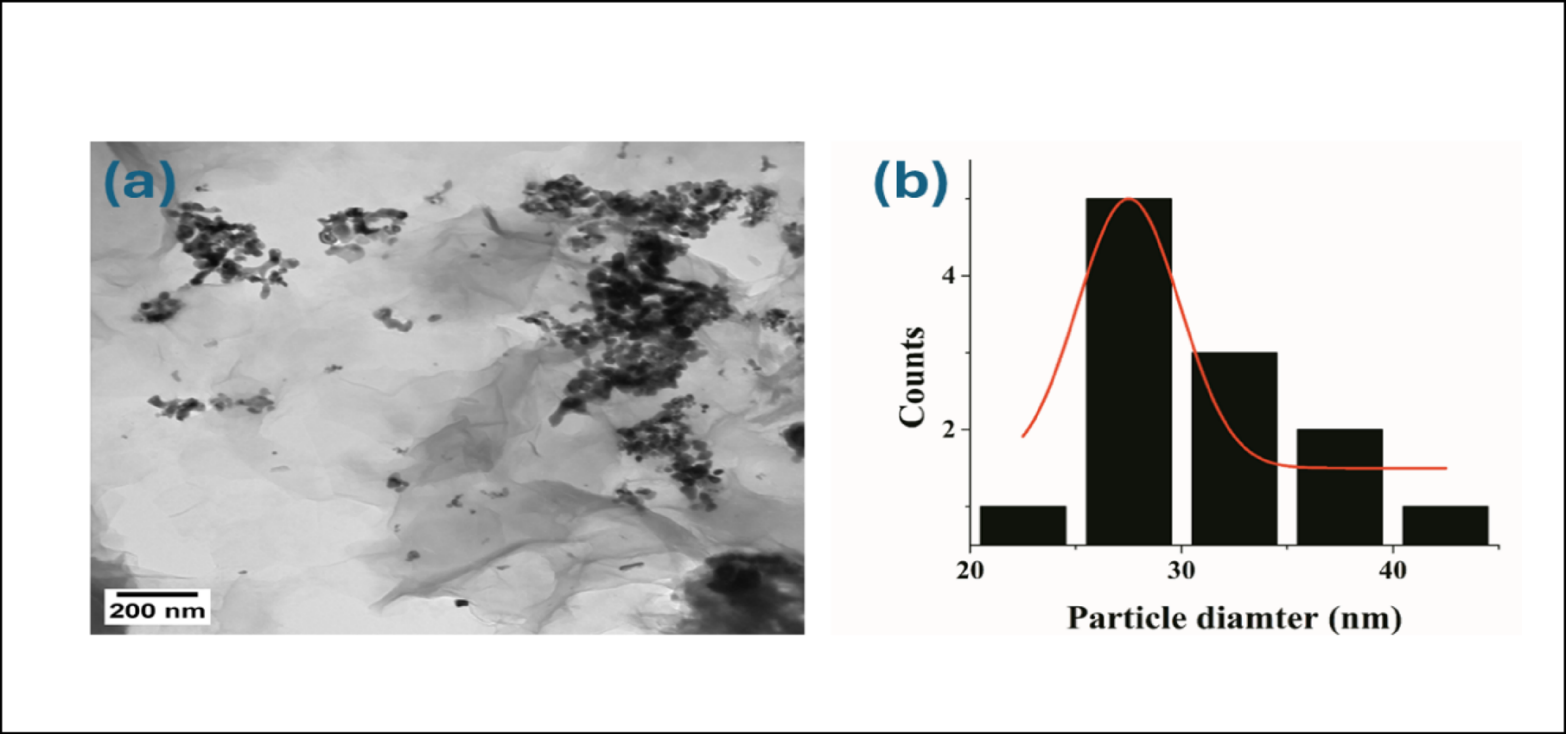
(**a**)TEM image for GO/NiO/CuO NPs, (**b**) relative size distribution histogram of GO/ NiO/CuO NPs.

TEM was performed for a suspension of GO/NiO/CuO NPs, where a beam of electrons interacted with the sample, transmitted through it, and the resulting image was magnified using a detector^67^. The average particle size of NiO/CuO NPs was approximately 27.5 nm. The histogram showed that a high percentage of the nanoparticles had diameters within the range of 27.5 ± 2 nm that is a key factor. Nanoparticles offer a much higher surface area, providing more active sites for electrochemical reactions at the interface and enhances the charge transfer kinetics. The distribution of NiO/CuO NPs on the surface of GO sheets further maximizes this effect, creating a highly accessible and porous structure. Figure 2a, b shows a TEM image and particle size histogram for NiO/CuO NPs, respectively.

**Fig. 3:**
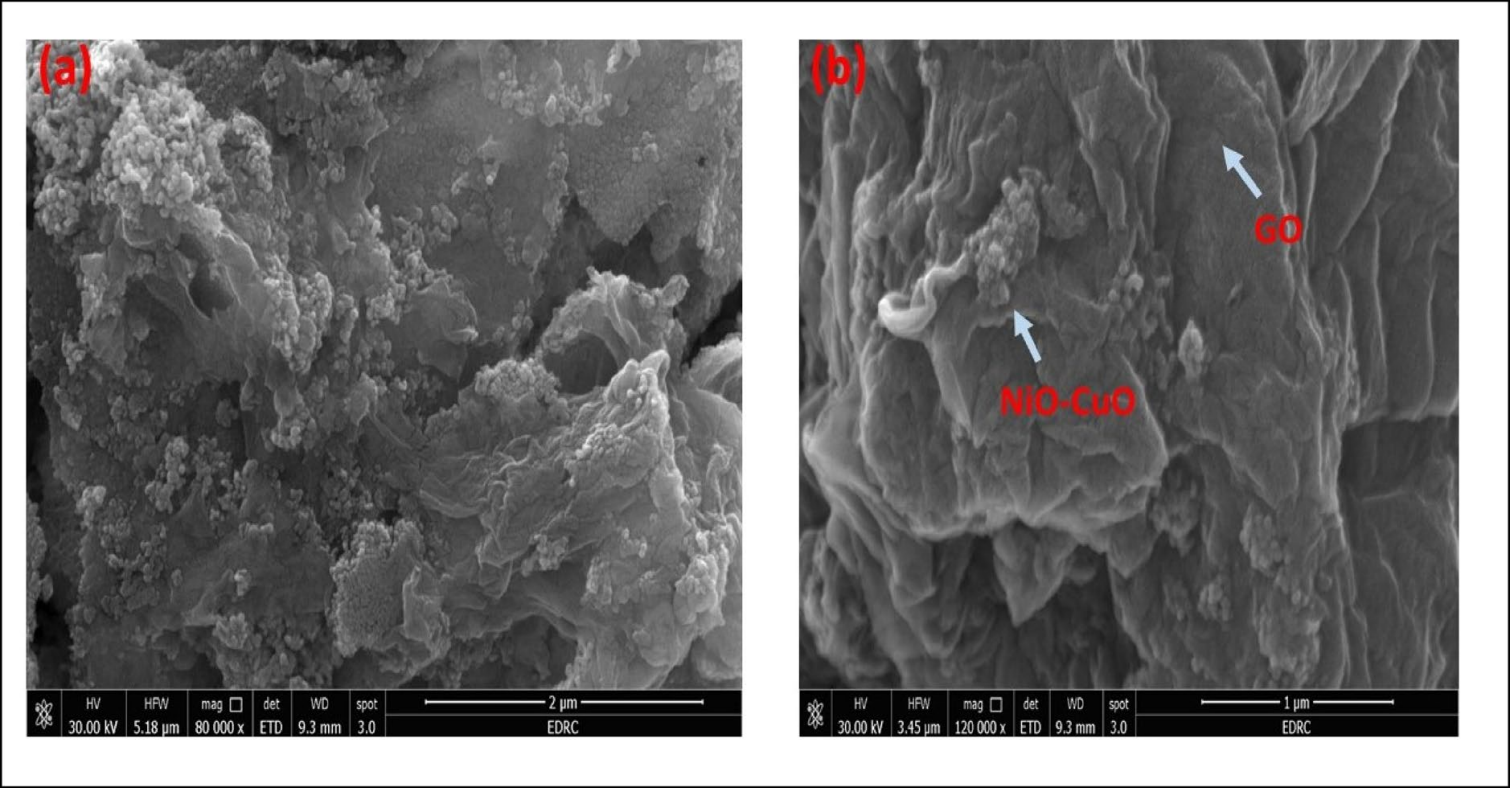
SEM analysis for GO/ NiO/CuO NPs.

**Fig. 4:**
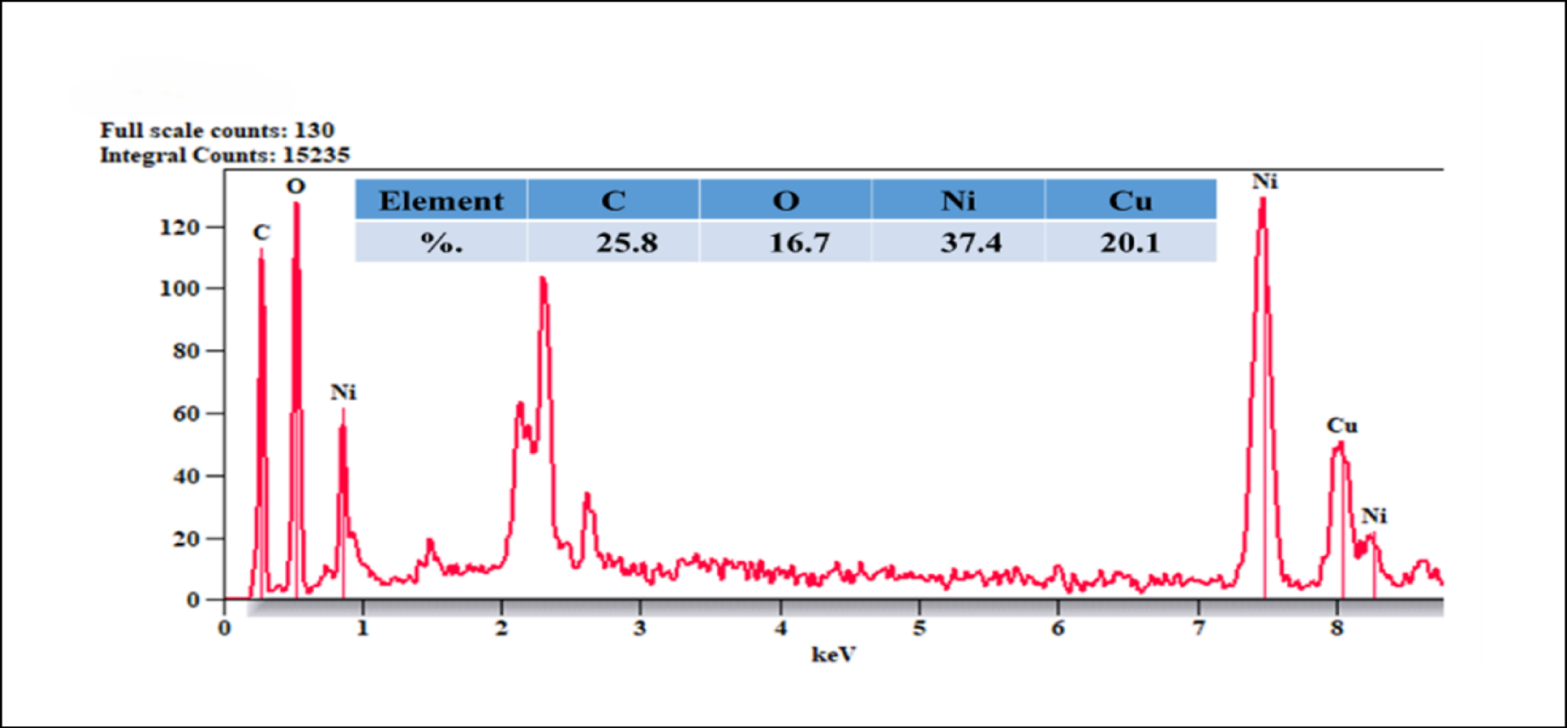
EDXA analysis of GO/NiO/CuO NPs.

**Fig. 5:**
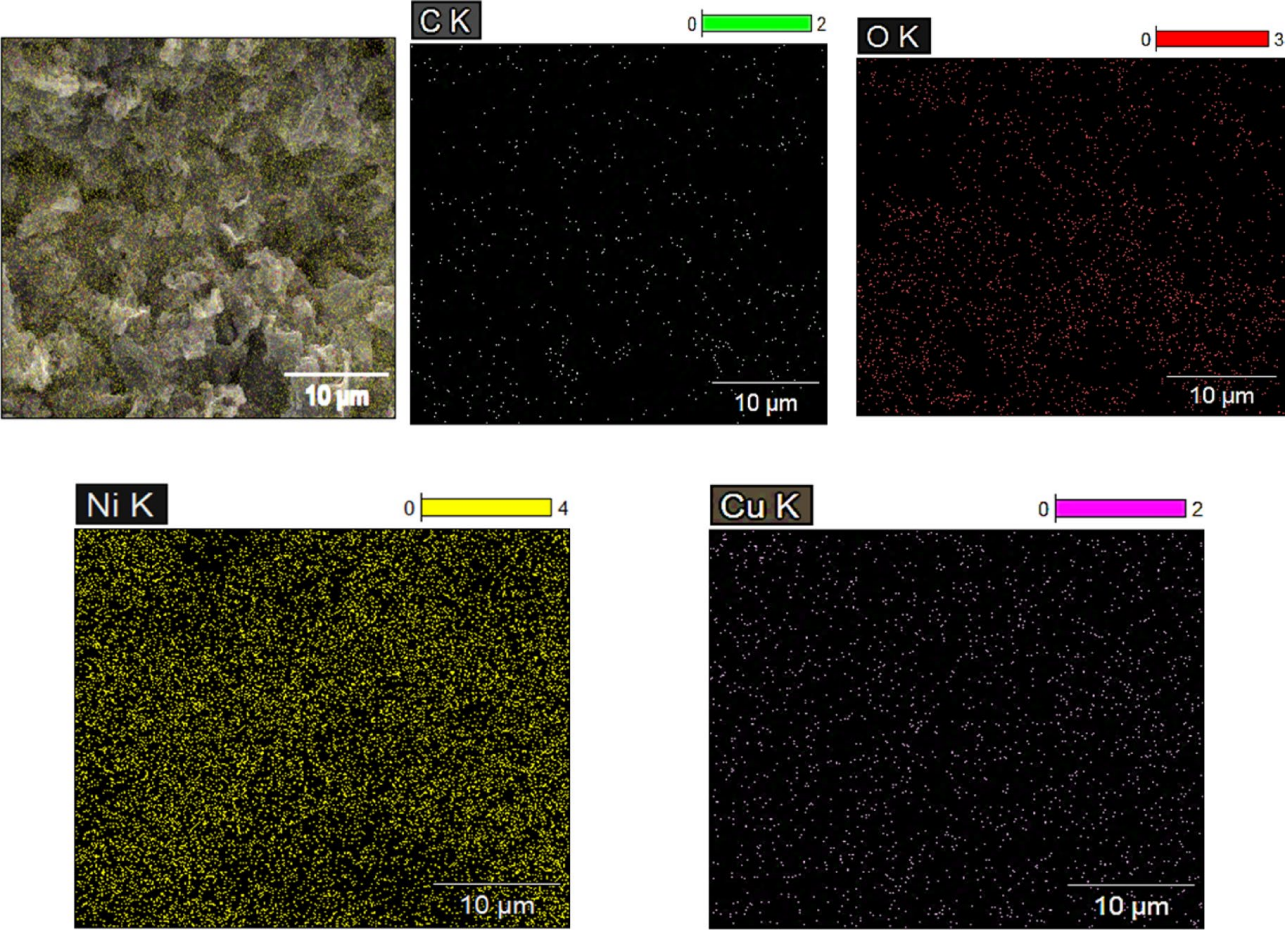
Mapping of EDXA analysis for GO/NiO/CuO NPs.

SEM analysis technique provided information about the surface morphology of GO/NiO/CuO NPs and identified electroactive sites on the surface that can undergo oxidation^67,68^. Figure 3 shows SEM images reveal the morphology of GO/NiO/CuO NPs as graphene oxide sheets with metals oxide nanoparticles distributed on their surface. SEM images confirm the uniform distribution of NiO and CuO nanoparticles on GO sheets, demonstrating their synergistic effect. GO provides a conductive, high-surface-area support that prevents agglomeration and enhances electron transfer, while the metal oxides nanoparticles provide active sites for fast ion diffusion and efficient charge transfer. EDXA, coupled with SEM, was used to obtain information about the surface composition and elemental analysis of the sample. EDXA results confirm the presence of carbon (C), oxygen (O), nickel (Ni), and copper (Cu), with their respective percentage quantities shown in Figure 4. Elemental mapping illustrates the spatial distribution of these elements, as shown in Figure 5.

### Electrochemical detection of sitagliptin phosphate monohydrate

**Fig. 6:**
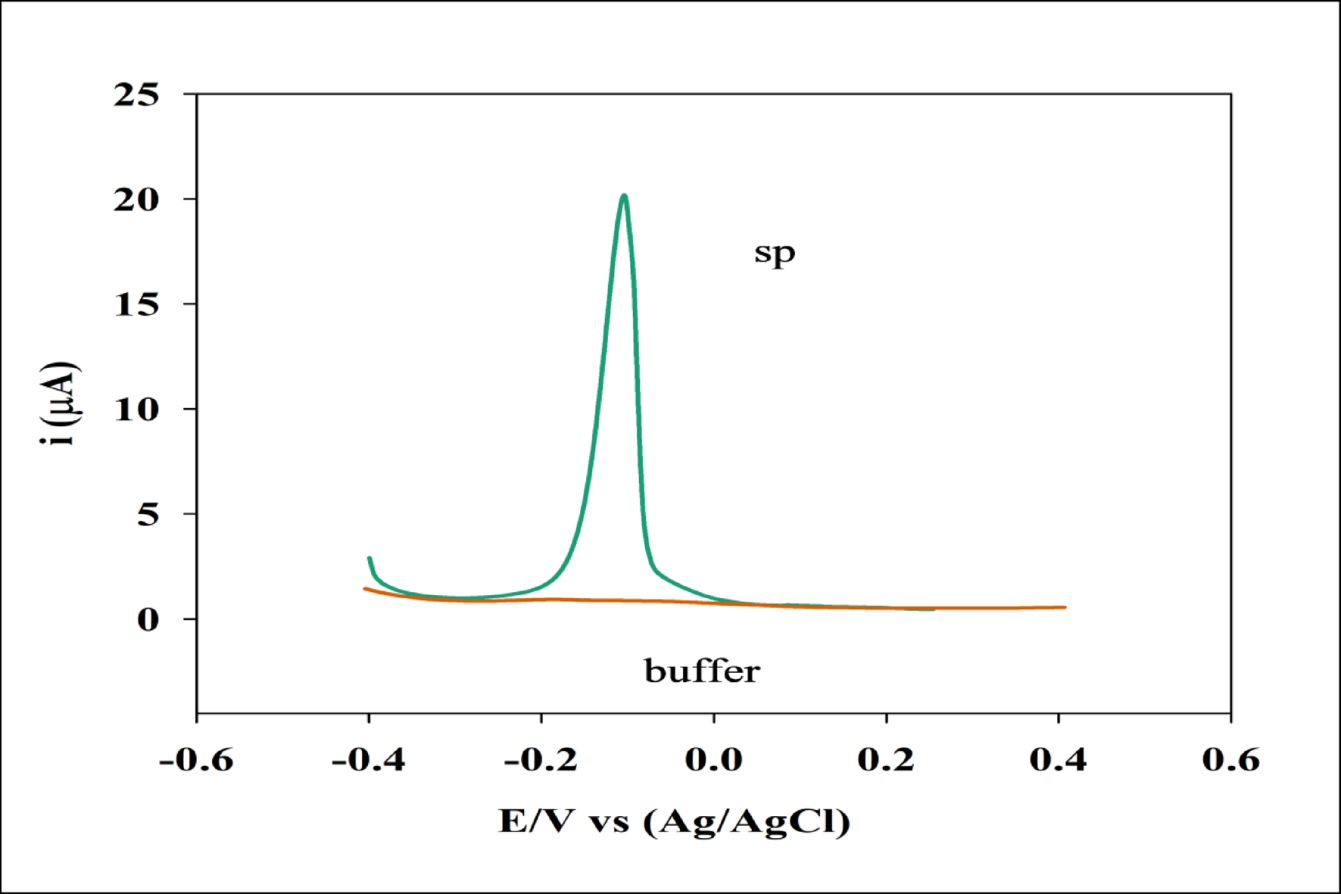
DPV response of sensor prepared using CME of GCE/GO/NiO/CuO NPs in case of PBS, pH 7.4, 0.1 mol/L and with addition of 1mM stock solution of SP. DPV parameters: potential range − 0.4: 0.4 V, modulation amplitude 50 mV, modulation time 50 ms.

The Voltammetric response of the proposed sensor, consisting of a GCE/GO/NiO/CuO NPs working electrode, was investigated toward SP using various techniques, including DPV, CV, and EIS, under optimized conditions at room temperature in a suitable PBS, pH 7.4, 0.1 mol/L. Voltametric response of the sensor was tested in the case of 10 mL PBS, pH 7.4, 0.1 mol/L and 1 mM stock solution of SP using DPV with parameters: potential range − 0.4 to 0.4 V, modulation amplitude 50 mV, modulation time 50 ms. As depicted in Figure 6, the sensor has no response toward the buffer used because PBS contains no electroactive species within the studied potential range. Thus, no redox activity occurs in the absence of SP but by the addition of SP solution, the response appeared at -0.1 V with a peak current (Ip) equal to 19.77 µA.

The electrochemical response of SP varied significantly with the nature of the modified electrode surface, as summarized in **Table S2**. The bare GCE exhibited no observable oxidation peak, indicating sluggish electron-transfer kinetics and poor affinity toward SP. Introducing graphene oxide onto the GCE markedly enhanced the oxidation current (6 µA) and enabled oxidation at a relatively low potential (− 0.12 V), reflecting the high surface area and abundant oxygenated functional groups of GO, which facilitate adsorption and electron shuttling.

A further improvement was achieved upon modification with NiO/CuO nanoparticles, where the oxidation current increased to 14 µA with a slight shift in oxidation potential to − 0.13 V. This enhancement can be attributed to the mixed-metal oxide’s redox-active nature, improved conductivity, and catalytic sites that promote the oxidative transformation of SP. Notably, the highest performance was recorded for the ternary composite GCE/GO/NiO/CuO NPs, which produced a substantially larger oxidation current (20 µA) accompanied by a more favorable oxidation potential (− 0.10 V). The synergistic interaction between GO sheets and binary metal-oxide nanoparticles likely increases the electroactive surface area, accelerates charge transfer, and provides abundant active centers for catalytic oxidation.

Using the Randles- Sevcik equation, the electroactive surface area (ECSA) was determined by CV in potassium ferrocyanide. For the Fe(CN$\:{)}_{6}^{-4}$ /Fe(CN$\:{)}_{6}^{-3}$ redox couple (*n* = 1), GO electrode exhibited an ECSA of about 0.131 cm², close to its geometric area. In contrast, NiO/CuO NPS composite reached 0.214 cm², reflecting its porous nanostructure and higher roughness factor, which provide more electroactive sites and superior charge-transfer capability.

#### Studying effect of scan rate using Cyclic voltammetry (CV)

**Fig. 7:**
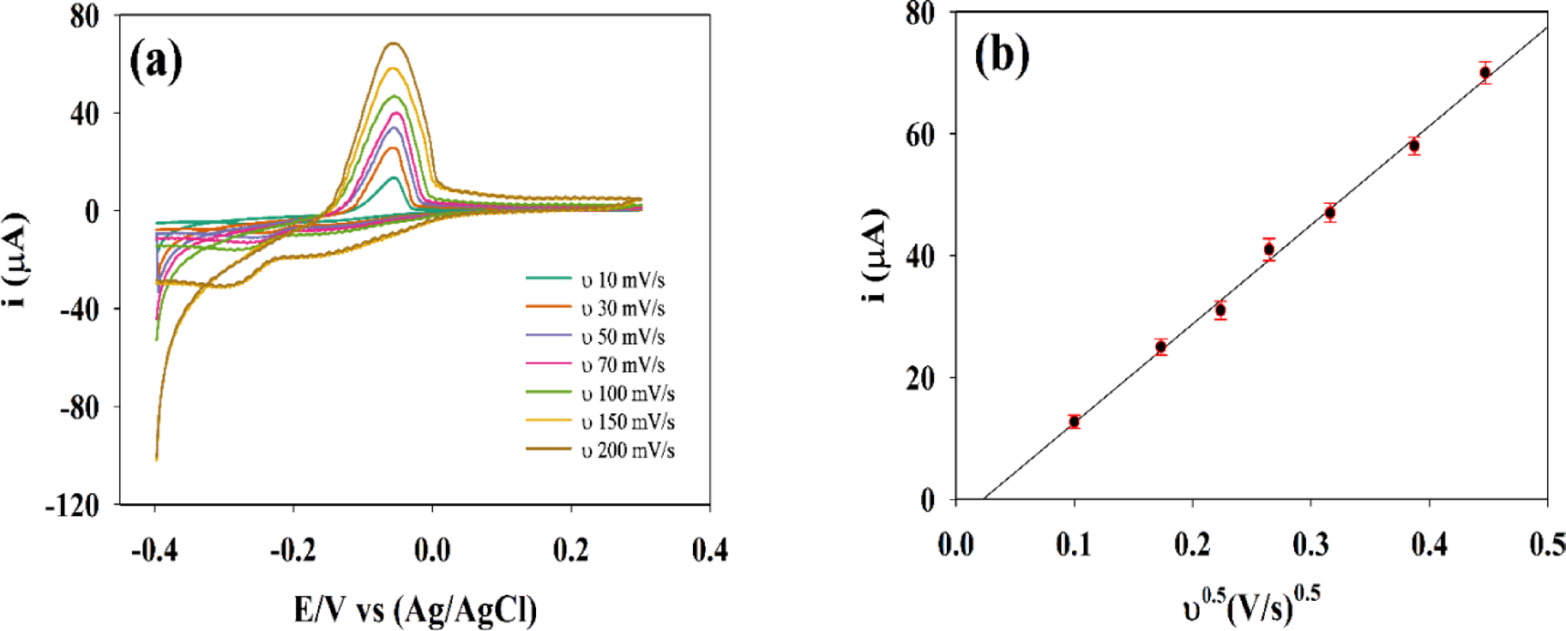
(**a**) CV voltammograms for 0.614 SP solution in PBS, pH 7.4, 0.1 mol/L in potential range − 0.4: 0.4 V at different scan rates 10, 30, 50, 70, 100, 150 and 200 mV/s. (**b**) Relation between Ip and $\:{{\upnu\:}}^{1/2}$. CV parameters: start potential − 0.4, end potential 0.4, number of cycles 10.

The first electrochemical technique applied was CV, a valuable method due to its various advantages, such as simple operation, assessing the reversibility of the oxidation-reduction reaction of the analyte, providing information about reaction mechanism and studying electron transfer kinetics^69^. Important parameters can be determined from CV voltammograms as redox potential and diffusion coefficients. Under optimized CV conditions, the electrochemical cell was assembled as described previously. The first step involved activating the buffer by applying a potential and measuring the current to ensure there was no electrochemical response from the buffer toward the prepared sensor GCE/GO/NiO/CuO NPs. A potential was then applied to 0.614 mM SP solution in PBS, pH 7.4, 0.1 mol/L at various scan rates (10, 30, 50, 70, 100, 150, and 200 mV/s). Voltammograms were obtained, with Ip observed at − 0.055 V, as shown in Figure 7a.

According to Randles - Sevick equation, Ip in CV increases linearly with $\:{{\upnu\:}}^{1/2}$ This behavior occurs due to a higher concentration gradient of the electroactive species near the electrode surface during the redox reaction. Therefore, increasing the scan rate of the applied potential results in a higher measured current. A relation between Ip and $\:{{\upnu\:}}^{1/2}$. was plotted as shown in Figure 7b. This equation is widely used to study whether the oxidation or reduction process is diffusion-controlled, by enabling the calculation of D of the electroactive species. At a standard temperature of 25 °C (298 K), the Randles-Sevcik equation simplifies to^70–74^:

1 $$\text{Ip}=2.69\times\:{10}^{5}\:A\:{\text{D}}^{1/2}\:{n}^{3/2}\:\:{{\upnu\:}}^{1/2}$$

Where Ip = peak current (A), ν = scan rate (V/s), n = number of electrons transferred, D = diffusion coefficient (cm²/s), F = Faraday constant (C/mol), R = universal gas constant (J/K·mol), T = temperature (K), A = electrode surface area (cm²), C = bulk concentration of analyte (mol/cm³).

This reveals a linear relationship between Ip and $\:{{\upnu\:}}^{1/2},$ where the slope of the line is:

2 $$\text{Slope}=2.69\times\:{10}^{5}\:A\:{\text{D}}^{1/2}\:{\text{n}}^{3/2}$$

Given that slope of linear relation = $\:1.55\times\:{10}^{-4},$ A= 0.214 cm², *n*= 2 and C= $\:6.14\times\:{10}^{-7}$ mol/cm³ the provided D for the modified electrode was estimated from a linear relation as $\:\:2.41\times\:{10}^{-6}$ cm²/s.

#### Calibration curve for SP using differential pulse voltammetry (DPV)

**Fig. 8:**
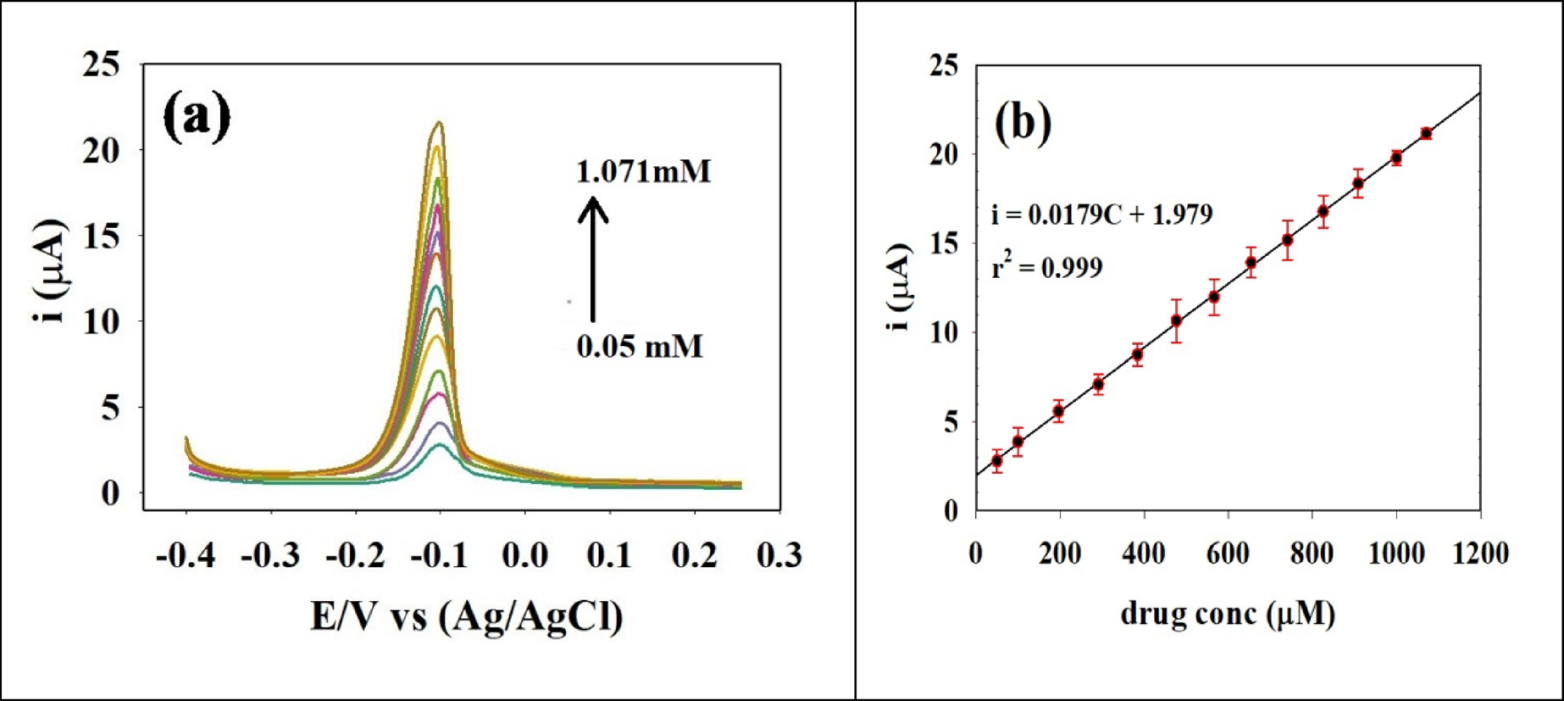
(**a**) DPV voltammograms for different concentrations of SP at optimized conditions PBS, pH 7.4, 0.1 mol/L. (**b**) Linear relation between Ip and concentration of SP. (DPV) parameters: potential range − 0.4 to 0.4 V, modulation amplitude 50 mV, modulation time 50 ms.

DPV was used to evaluate the sensitivity and selectivity of the prepared sensor toward SP, as it is a highly sensitive and quantitative technique in electrochemical analysis that minimizes the influence of capacitive current on the faradaic current^75^. DPV can achieve lower detection limits, thus has an advantage in the detection of trace levels of analytes. Common parameters, such as peak potential, analyte concentration and kinetics can be evaluated using DPV, thus determining the performance of modified electrodes. Response of SP to an oxidation-reduction reaction was at -0.1 V using CME of GCE/GO/NiO/CuO NPs as the working electrode versus Ag/AgCl /sat. KCl as a reference electrode in PBS, pH 7.4, 0.1 mol/L, with stirring. Under optimized DPV conditions, various concentrations of SP were analyzed, and the corresponding voltammograms were obtained, as shown in Figure 8a. A linear calibration curve for SP was constructed over the concentration range of 0.05 to 1.071 mM. LOD and LOQ were determined to be 0.0223 mM and 0.0677 mM, respectively using linear relationship observed between Ip and SP concentration as shown in Figure 8b, with a slope (S) of 0.0179, an intercept (b) of 1.979, and a correlation coefficient (r²) of 0.99. The standard deviation (σ) was calculated to be 0.1213. the sensitivity of the electrode was estimated as 0.0179 µA/µM.

LOD and LOQ were calculated as follows^76–81^:

3 $$LOD = 3.3 \sigma ​/S$$

4 $$LOQ = 10 \sigma ​/S$$

#### Electric impedance spectroscopy (EIS)

EIS is a powerful electroanalytical technique that investigates properties at the interface between the reference electrode and the working electrode CME, such as charge transfer, diffusion processes, and solution resistance. These properties are commonly represented using an electrical model called an equivalent circuit^82,83^. EIS has high sensitivity to surface modifications and ability to study electrochemical activity over a wide range of frequencies. An equivalent circuit model was used to interpret electrochemical parameters from the EIS measurements, including the charge transfer resistance, solution resistance, double-layer capacitance, and diffusion-related behavior. Subsequently all these parameters can be evaluated making EIS an essential tool for overall performance of electrochemical sensors.

**Fig. 9:**
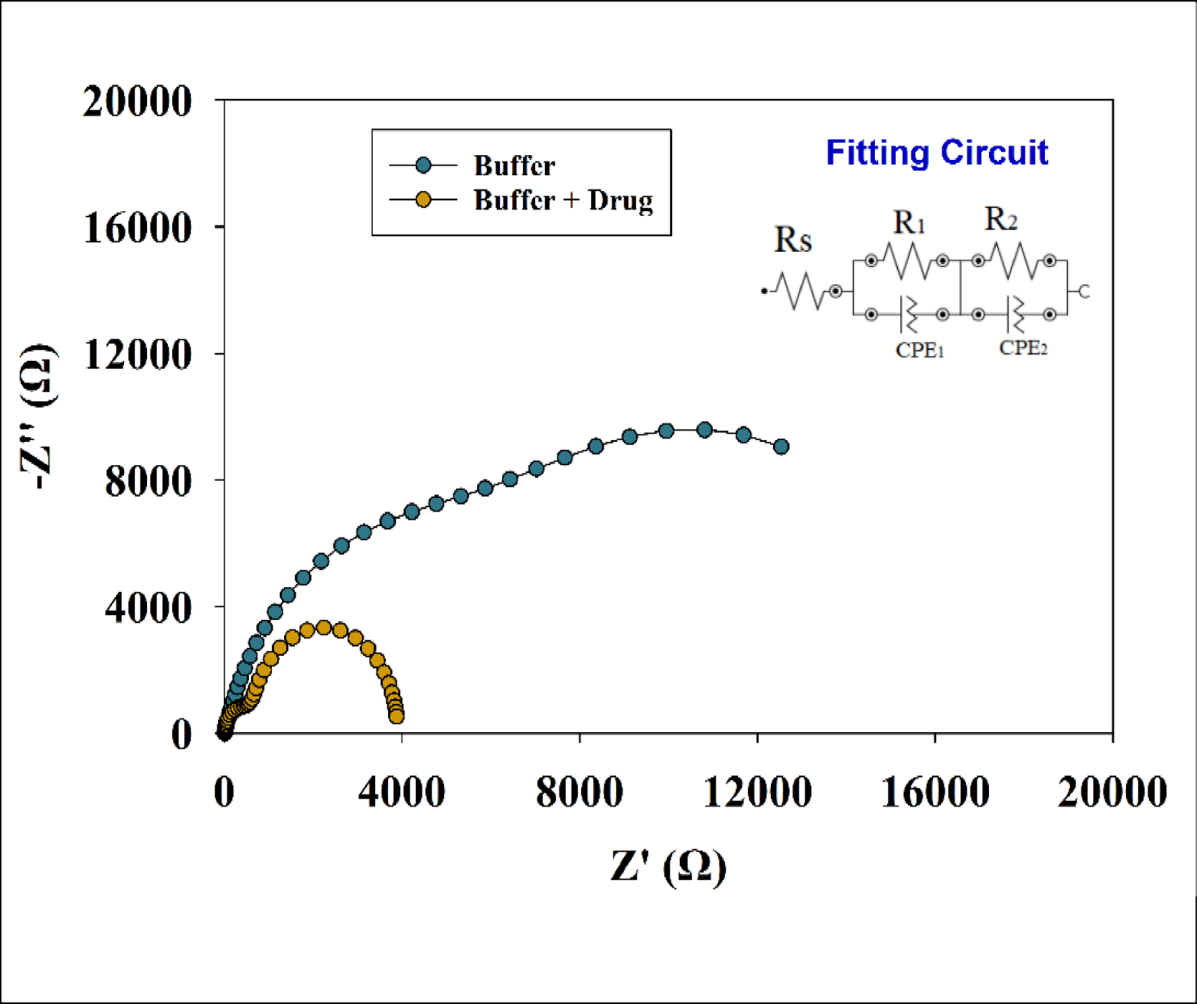
Nyquist plots of EIS for the GCE/GO/NiO/CuO NPS electrode in the presence of SP 1mM in PBS, pH 7.4, 0.1 mol/L at constant potential of 0.1 V (vs. Ag/AgCl sat. KCl) and absence of SP. Figure provided with fitting circuit for EIS data of SP.

EIS was performed using a potentiostat over a frequency range from 10 mHz to 10⁴ Hz, with a constant AC amplitude of 0.1 V . The Nyquist plot was obtained for the working electrode GCE/GO/NiO/CuO NPs versus a reference electrode Ag/AgCl/saturated KCl, using a platinum wire as the counter electrode. The measurements were performed for a 1 mM solution of SP in PBS, pH 7.4, 0.1 mol/L. The Nyquist plot, shown in Figure 9, displayed two partially overlapping semicircles, indicating two distinct electrochemical processes. The corresponding equivalent circuit consisted of the solution resistance (Rs) connected in series with two parallel RC elements. Each RC element included a charge transfer resistance (R₁ or R₂) connected in parallel with a constant phase element (CPE₁ or CPE₂), which represents the non-ideal double-layer capacitance. The high-frequency semicircle, represented by R₁ and CPE₁ and typically located on the left side of the Nyquist plot, corresponds to surface/interface effects such as charge transfer at the electrode/electrolyte interface. In contrast, the low-frequency semicircle, represented by R₂ and CPE₂ and usually found on the right side of the plot, corresponds to slower processes such as diffusion or bulk-related phenomena. Here, R₁ and CPE₁ correspond to the outer layer, while R₂ and CPE₂ represent the inner layer of the electrode interface. Figure 9 shows both the Nyquist plot and the fitted equivalent circuit. The fitted parameter values are listed in Table 1 and show a decrease in Rs, R₁, and R₂ in the presence of the drug SP compared to the buffer-only condition. Meanwhile, the values of CPE₁ and CPE₂ increased, which is attributed to enhanced electrochemical activity at the surface of the CME. This activity leads to greater current flow and a reduction in the diameter of the semicircles in the Nyquist plot.

**Table 1:** EIS fitting circuit parameters in case of buffer only or buffer with drug SP.

Electrolyte	Rs	R1	CPE1		R2	CPE2	
	Ohm	Ohm	*n*	Y0	Ohm	m	Y0
Buffer	17.15	5480	0.819	0.00010034	14,601	0.823	0.0001898
Buffer + Drug	15.2	560	0.775	0.0001818	3896	0.786	0.0002489

#### Application in real samples

**Fig. 10:**
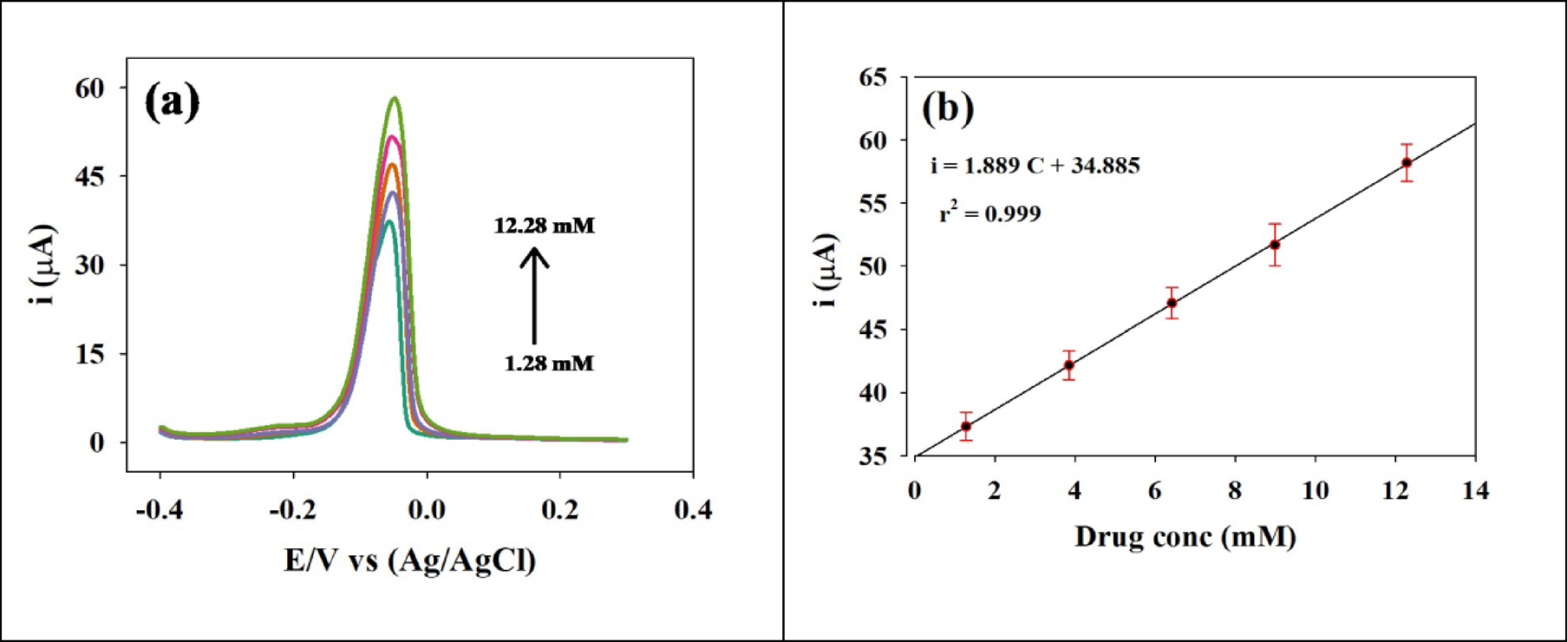
(**a**) DPV voltammograms for different concentrations of SP real tablet in PBS, pH 7.4, 0.1 mol/L. (**b**) Linear relation between Ip and concentration of SP in tablet form. DPV parameters: potential range − 0.4 to 0.4 V, modulation amplitude 50 mV, modulation time 50 ms.

Sensor sensitivity toward Januvia tablet STG 50 mg, which contains approximately 64.25 mg of SP, was investigated using DPV. Notably, Januvia tablets contain several inactive ingredients that may influence the sensor’s current response to SP. DPV was performed on various concentrations of SP real tablets after dilution. Figure 10a shows voltammograms of SP tablet in the presence of excipients. A linear relationship between Ip and sp concentration was observed as shown in Figure 10b and regression data are provided in Table 3. A noticeable positive shift in potential was observed at -0.05 V, compared to -0.1 V in the case of SP powder, along with an increase in Ip. This shift is likely due to the influence of the inactive ingredients present in the tablets. The sensitivity was provided as 0.01889 µA/µM.

**Fig. 11:**
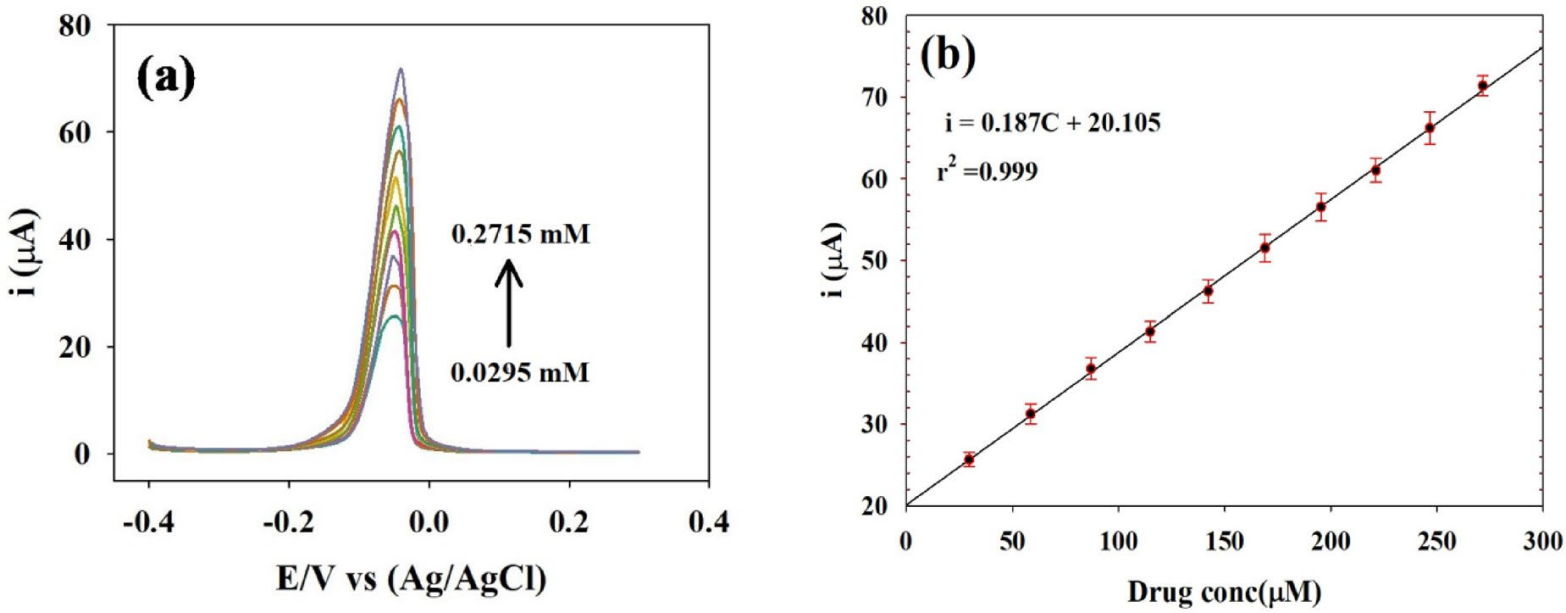
(**a**) DPV voltammograms for different concentrations of SP in spiked plasma sample in PBS, pH 7.4, 0.1 mol/L. (**b**) Linear relation between Ip and concentration of SP in spiked plasma. DPV parameters: potential range − 0.4 to 0.4 V, modulation amplitude 50 mV, modulation time 50 ms.

The determination of SP in biological fluids is a critical process for studying the pharmacokinetics of the drug. According to the prescribing information for Januvia, peak plasma concentrations of SP occur within 1 to 4 h after administration, and the drug is primarily excreted via the urine. Under optimized DPV conditions, various concentrations of SP were analyzed by repeatedly adding 100 µL aliquots of a 3 mM standard SP solution to spiked plasma solution. Voltammograms and calibration curves were obtained, as shown in Figure 11a. As shown in Figure 11b a linear relation between Ip and SP concentration in spiked plasma was constructed over the concentration range of 0.0295–0.2715 mM. LOD and LOQ were determined to be 0.0061 mM and 0.0185 mM, respectively and regression data are provided in Table 3. The results indicated an increase in Ip, likely due to the presence of electrolytes in the spiked plasma, with a response observed at -0.05 V. The sensitivity of the electrode was found to be 0.187 µA/µM.

### Comparison of electrochemical performance

As shown in Table 2. various electrochemical approaches have been employed for sitagliptin analysis, including potentiometry and voltammetry (e.g., DPV and LSV). The design of the working electrode is a critical factor influencing the final electrochemical performance. For working electrode materials and modifications with the exception of the traditional modified carbon paste electrode used in study^18^, the majority of the reported sensors utilize non-traditional, complex modification strategies. Specifically, the study used pt electrode modified with electroactive molecularly imprinted nanoparticles (nanoMIP), which involves complex specialized synthesis and characterization steps^19^. Similarly, potentiometric sensor in study^21^ relies on sophisticated and time-consuming preparation of a Molecularly Imprinted Polymer (MIP). In contrast, our work introduces a novel composite material (GCE/GO/NiO/CuO NPS) which offers a simpler, yet highly effective alternative based on easily synthesized nanomaterials and their catalytic effects. Analytical Technique that required definite preparation methods as study employed Linear Sweep Voltammetry (LSV) on a renewable amalgam film electrode (Hg (Ag)FE)^20^. The preparation and renewal of such amalgam film electrodes, often require specific, sensitive procedures and strict control of experimental conditions, increasing the complexity of the routine assay. In our study we used DPV with (GCE/GO/NiO/CuO NPS) so allows for superior sensitivity due to background current reduction, but on an easily prepared and robust solid electrode, significantly simplifying the analytical process for routine use.

Sensitivity is often the most important factor to investigate different electrodes performance however some methods, particularly the Pt nanoMIP sensor^19^, achieve ultra-trace detection limits LOD of 6 pM, they often sacrifice simplicity. Our study’s LOD achieved in the spiked plasma sample LOD of 0.0061 mM that demonstrates its direct applicability and sufficient sensitivity for pharmacological quantification. This is achieved with an electrode that is easier to fabricate and maintain than those utilizing MIP or mercury-based systems^20,21^.

**Table 2:** Comparison of electrochemical performance of different electrodes for sitagliptin detection.

Analyte	LOD	Technique	Electrode Type & Modification	Sample	Ref	Date
Sitagliptin	9.1 nM	potentiometry	Carbon paste electrode with phosphotungstate	Real samples	^18^	2022
Sitagliptin	0.06 pM	Voltammetry (DPV)	Pt electrode/nanoMIP sensors	Plasma	^19^	2021
Sitagliptin	2.6 nM	linear sweep voltammetry (LSV)	amalgam film electrode Hg (Ag)FE	Pharmaceutical product	^20^	2020
Sitagliptin	2.6µM	potentiometry	Potentiometric sensors incorporated with molecular imprinted polymer (MIP)	pharmaceutical formulations and biological fluids	^21^	2014
Sitagliptin phosphate monohydrate	0.0223 mM powder	Voltammetry (DPV)	GCE/GO/NiO/CuO NPs	Powder Tablet spiked plasma	This study	---
	0.22 mM Tablet					
	0.0061 mM spiked plasma					

### Method validation by detection of SP using HPLC-UV technique

**Fig. 12:**
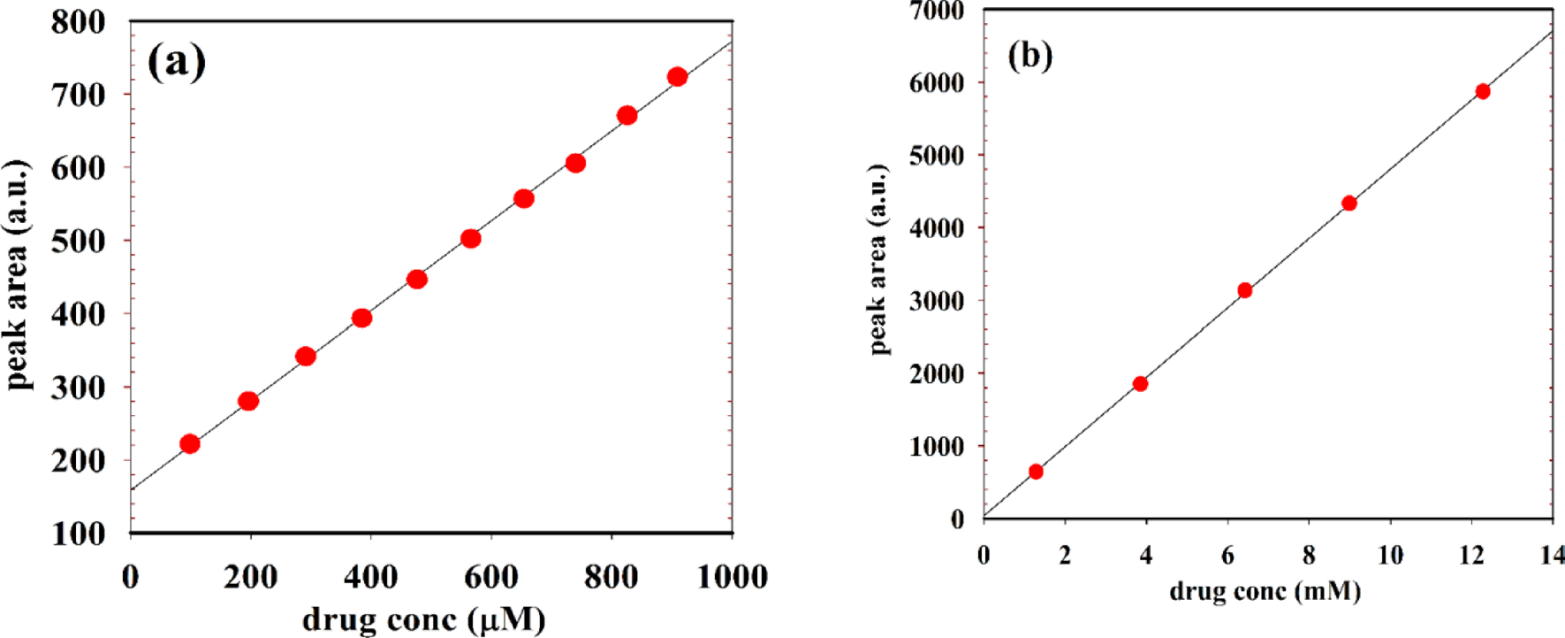
calibration curves of SP using HPLC–UV at optimized conditions. (**a**) powder form, (**b**) tablet.

HPLC analysis was performed to detect SP in both pure powder form for research purposes and in commercial tablet form for method validation. The results obtained from the HPLC method were compared with those from the proposed electrochemical method described in this study. the HPLC system used was an Agilent 1200 Series HPLC system (Agilent Technologies, Santa Clara, CA, USA) equipped with a UV detector. The stationary phase consisted of either an Inertsil ODS-4 or Agilent Eclipse Plus C18 column (250 × 4.6 mm, 5 μm particle size). The injection volume was 15 µL, and the column temperature was maintained at 40 °C. UV detection was carried out at a wavelength of 266 nm. The mobile phase consisted of acetonitrile and PBS (pH 3.0) in a 35 to 65 (v/v) ratio, with a flow rate of 1.0 mL/min. The retention time of SP was observed between 3.15 and 3.16 min, with a total analysis time of approximately 5 min. A sequence summary report for HPLC-UV analysis of SP tablets, including retention times, peak heights, and peak areas at various concentrations, is provided in the supplementary file. Calibration curves were constructed for both the pure powder and tablet forms to evaluate the linear relationship between peak area and SP concentration. The sample solutions were prepared following the same procedure and concentration ranges as used in the electrochemical method. As shown in Figure 12(a-b), linear relationship was observed in both cases. For the pure powder form, the concentration range was 0.099–0.9091 mM, with LOD of 0.0275 mM and a LOQ of 0.0834 mM. For the tablet form, the concentration range was 1.28–12.28 mM, with an LOD of 0.1922 mM and an LOQ of 0.583 mM.

### Statistical analysis of results

#### Recovery (accuracy) calculations and linear regression data

Recovery calculations for the determination of SP using the electrochemical sensor based on GCE/GO/NiO/CuO NPs were performed to evaluate the efficiency and validate the proposed method. The results were compared with those obtained from a reported HPLC method. Calibration curves for SP analysis in pure powder, tablet, and spiked plasma were constructed, and linear relationships were established. Parameters including S, b, r², LOD, LOQ and σ are represented in Table 3. All recovery data for both methods are summarized in Tables 4 and 5, and the values were found to be close to 100%, confirming the accuracy and reliability of the electrochemical method.

The recovery (%) was calculated using the following equation:

5 $$Recovery (\%) = (Actual \;concentration / Theoretical \;concentration) \times 100$$

Where the actual concentration (**C**) was determined using the equation:

6 $$C = (Ip \;or\; A -- Intercept) / Slope$$

Here, **Ip** or **A** represents the peak current (in electrochemical methods) or absorbance (in spectrophotometric methods), respectively.

**Table 3:** Linear regression data for determination of SP by electrochemical and HPLC-UV methods in different drug forms.

Method & drug form	Linear relation	Linear range & LOD & LOQ	S	b	*r* ^2^	σ
Electrochemical (powder)	I = 0.01791 C + 1.979	0.050-1.071mM 0.0223&0.0677 mM	0.0179	1.979	0.999	0.1213
Electrochemical (Januvia tablet)	I = 1.889 C + 34.885	1.28–12.28 mM 0.22 &0.667 mM	1.889	34.885	0.999	0.126
Electrochemical (powder in spiked plasma)	I = 0.187 C + 20.105	0.02956–0.2715 mM 0.0061 &0.0185 mM	0.187	20.105	0.999	0.346
HPLC-UV (powder)	A = 0.6141 C + 158.342	0.099–0.9091 mM 0.0275 &0.0834 mM	0.614	158.341	0.999	5.1213
HPLC-UV (Januvia tablet)	A = 476.491 C + 38.564	1.28–12.28 mM 0.192&0.583 mM	476.5	38.563	0.999	27.762

**Table 4:** Recovery calculations for calibration curves for determination of SP in powder and tablet form by electrochemical sensor (GCE/GO/ (NiO/CuO NPs)) and HPLC -UV.

Theoretical conc (mM)		Electrochemical sensor (GCE/GO/ NiO/CuO NPs)			HPLC-UV method		
		Current (µA)	Actual conc(mM)	Recovery (%)	Peak area	Actual conc(mM)	Recovery (%)
0.05	**Sitagliptin phosphate mono hydrate in powder form**	2.7829	0.0453	91	---	---	---
0.099		3.8500	0.1049	105.9	221.596	0.10302	104.06
0.196		5.58	0.20156	102.8	280.121	0.19833	101.18
0.291		7.089	0.2859	98.24	341.504	0.29831	102.51
0.384		8.7349	0.3778	98.38	393.283	0.38264	99.64
0.476		10.6390	0.48418	101.7	446.265	0.46893	98.51
0.566		11.9644	0. 55,823	98.63	501.958	0.55963	98.87
0.654		13.9007	0.66641	101.89	556.791	0.64894	99.22
0.740		15.16	0.73676	99.56	605.254	0.7278	98.35
0.825		16.78	0.82726	100.27	670.799	0.08346	101.16
0.909		18.356	0.91531	100.69	723.591	0.9206	101.27
1		19.779	0.99480	99.48	---	---	---
1.071		21.1508	1.07144	100.04	---	---	---
1.28	**Sitagliptin phosphate mono hydrate in tablet form**	37.3254	1.2919	100.9	643.477	1.2695	99.18
3.85		42.1722	3.8577	100.2	1850.395	3.8024	98.76
6.42		47.0703	6.45066	100.47	3134.214	6.4967	101.19
8.99		51.6860	8.89412	98.93	4331.833	9.0102	100.22
12.28		58.1878	12.3360	100.45	5871.34	12.2411	99.68

**Table 5:** Recovery calculations for calibration curve for determination of SP by electrochemical sensor (GCE/GO/ (NiO/CuO NPs)) in spiked plasma.

Current (µA)	Theoretical conc (mM)	Actual conc(mM)	Recovery (%)
25.6489	0.02956	0.02969	100.43
31.2451	0.05854	0.05966	101.91
36.7812	0.08696	0.08932	102.71
41.3422	0.11483	0.11375	99.05
46.2368	0.14218	0.13996	98.43
51.5215	0.16901	0.16827	99.56
56.5277	0.19535	0.19508	99.86
61.0181	0.2212	0.21913	99.06
66.224	0.24658	0.24702	100.17
71.3745	0.2715	0.27461	101.14

#### Repeatability and reproducibility studies (precision)

Both repeatability and reproducibility studies were performed to evaluate the precision of the method, and the relative standard deviation (RSD) values were found to be less than 1.5%. as shown in **Table S3 and S4** respectively. Inter-day stability was assessed at two time points (1st and 10th day). The oxidation peaks in both curves displayed consistent current responses with some decrease in oxidation current, confirming that the modified electrode retained stable electrochemical activity throughout the 10-day period. While a slight difference in peak intensity was observed, the variation was minimal, indicating that the electrode surface remained robust with no significant degradation or fouling over time (please see **Figure S3**).

### Selectivity and sensitivity of sensor toward SP in the presence of interferents

**Fig. 13:**
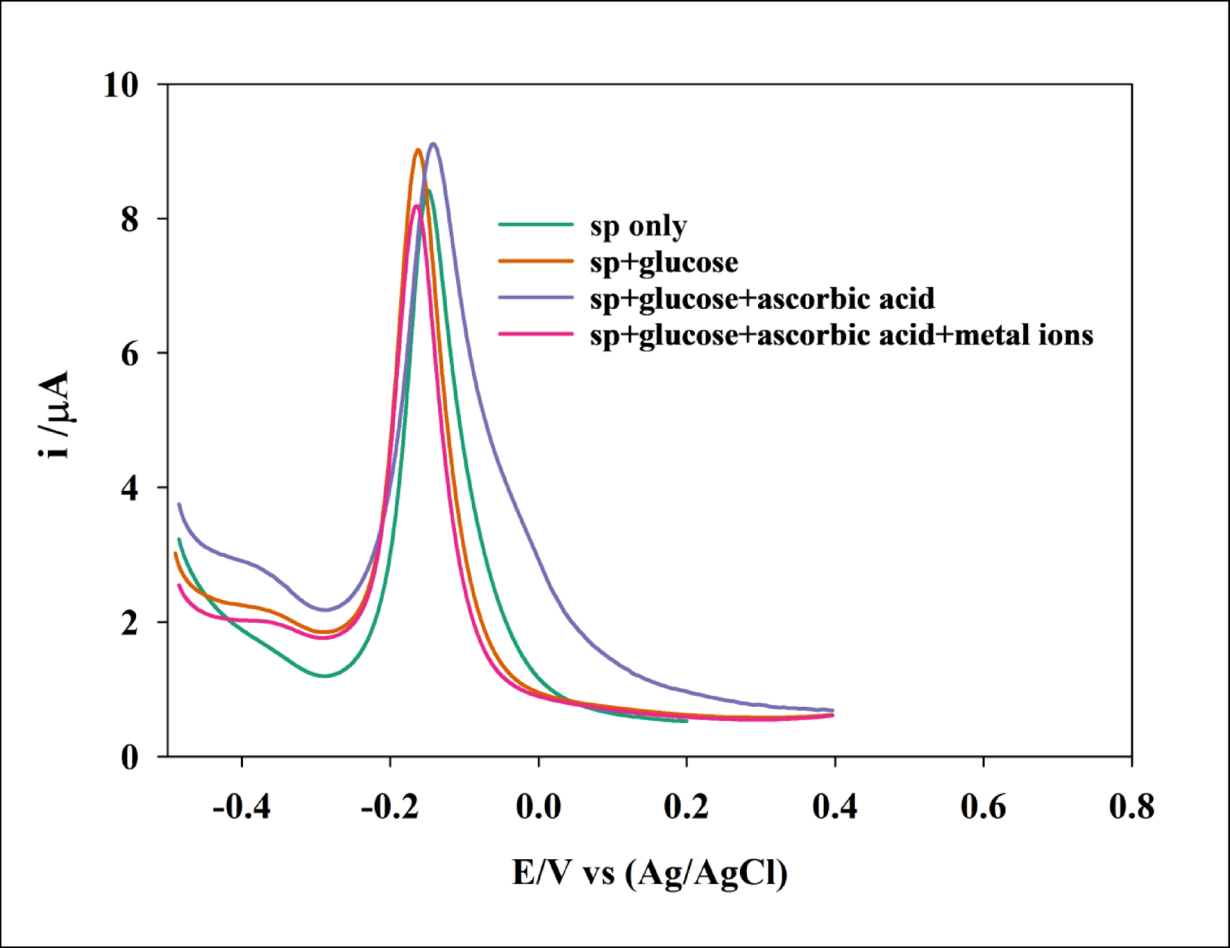
DPV voltammograms for SP in presence of interferents. DPV parameters: potential range − 0.4: 0.4 V, modulation amplitude 50 mV, modulation time 50 ms.

The selectivity and sensitivity of the sensor prepared using CME of GCE/GO/NiO/CuO NPs toward SP were studied using the DPV technique under optimized conditions, in the presence of various interferents such as glucose, ascorbic acid, and metal ions (Na₂SO₄, CuSO₄, and KCl). This was done to investigate any potential effects on the voltage response or Ip for a defined concentration of SP. DPV measurements were performed on a 0.4 mM SP solution in PBS, pH 7.4, 0.1 mol/L, both alone and in the presence of the interferents. The resulting voltammograms are shown in Figure 13. As seen in Table 6, the voltage response of the sensor toward SP was not significantly affected, and Ip showed only a minor increase of less than 1 µA. These results demonstrate that the sensor exhibits good selectivity and sensitivity toward SP.

**Table 6:** Shows voltage response and peak current for DPV voltammograms for SP in presence of interferents.

analyte		Voltage response (V)	Peak current (µA)
1	sp only	-0.1475	8.4073
2	sp + glucose	-0.162	9.023
3	SP + glucose + ascorbic acid	-0.1425	9.11
4	SP + glucose + ascorbic acid + metal ions	-0.162	8.1756

## Conclusion

The proposed method for designing the GCE/GO/ (NiO/CuO NPs) sensor is simple, highly sensitive, and selective toward sitagliptin phosphate monohydrate (SP), whether in powder, tablet form, or biological fluid as spiked plasma. This is attributed to the excellent properties of the CME components, particularly the GCE, which offers high electrical conductivity, stability, and chemical inertness. The use of nanomaterials such as GO/ NiO/CuO NPs enhanced the sensor’s performance due to their high surface area, catalytic properties, and efficient electron transfer capability, all of which are advantageous for electrochemical reactions. Various electrochemical techniques were employed with this sensor to detect SP, and the recovery results were close to 100% while RSD values were less than 1.5%. The method was validated by HPLC analysis for both powder and tablet forms within the same concentration ranges. This method offers simplicity in design, versatility in performance allowing drug detection in different forms and in biological fluids as spiked plasma, with or without interferents and ease of material preparation. Compared to previous electrochemical methods for detecting sitagliptin phosphate monohydrate, this approach represents a significant advancement.

## Supplementary Information

Below is the link to the electronic supplementary material.

Supplementary Material 1

## Data Availability

All data generated or analysed during this study are included in this published article [and its supplementary information files].
