# Supplementary material for: NiO/CuO@Graphene oxide-modified electrode for sensitive detection of an antidiabetic drug
Source: Sci Rep. 2026 Jan 6;16:806. doi: 10.1038/s41598-025-32562-1 (PMC12780167; doi:10.1038/s41598-025-32562-1)
Supplement: Supplementary file 1 — Supplementary Material 1 [file 41598_2025_32562_MOESM1_ESM.docx]

**Supplement for: NiO/CuO@Graphene Oxide-Modified Electrode for Sensitive Detection of an Antidiabetic Drug.**

**Essam N. Labeeb, Mahmoud A. Hefnawy*, Shymaa S. Medany, Eman Yossri Frag**

**Chemistry Department, Faculty of Science, Cairo University, 12613 Giza, Egypt**

***Corresponding author: Mahmoud A. Hefnawy ([maadel@cu.edu.eg](mailto:maadel@cu.edu.eg);** [**maahefnawy@gmail.com**](mailto:maahefnawy@gmail.com)**)**

**Table (S1) illustrates previous methods reported for determination of (SP) simultaneously or in combination with other antidiabetic drugs.**

| **Detection method** | **Drug** | **Linear dynamic range** | **Limit of detection LOD** | **Limit of quantization LOQ** | **RSD%** | **Accuracy%** | **sample** | **R^2** | **Date** | **REF NO** |
| --- | --- | --- | --- | --- | --- | --- | --- | --- | --- | --- |
| UV-VIS at 267nm | SP | 20-60 µg/mL | 6.03 µg/ml | 18.28µg/ml | 0.32 | 99.62%:  100.48% | Pure&  tablet | 0.991 | Dec  2012 | [1] |
| Colorimetric  -Visible spectrum  at 543 | Sitagliptin | 0.1-10 µg/mL | 0.03 mg/L | 0.1 mg/L | 1.03% | 98.98%  100.41% | Pharma- form | 0.9983 | Jan 2021 | [2] |
| Spectrofluori-metric | Sitagliptin phosphate | 0.1- 2 mM | 3.7$\times$10^-4^ mM | 1.23$\times$10^-3^ mM | --- | 97.41% -  103.36 % | Pharma.  prep&  Bio-fluids | 0.9997 | July  2023 | [3] |
| HPLC-UV  detector  at 254nm | Sitagliptin  Metaformin  atorvastatin | 3.125-100 μg/ml SIG  0.625-25 μg/ml MF  0.3125-10μg/ml AV | 0.82 μg/mL SIG  1.2 μg/mL MF  0.09 μg/mL AV | 2.46 μg/mL SIG  1.2 μg/mL MF  0.27 μg/ml AV | --- | --- | pure form  &  Ph. Form | 0.9976  SIG  0.9995  MF  0.9994  AV | 2014 | [4] |
| RP-UPLC  Detection at  218 nm | Sitagliptin  Ertugliflozin | 25-125  µg/mL SIT  3.75-22.5  µg/ml ERT | 0.48 µg/mL  SIT  0.11 µg/mL ERT | 1.46 µg/mL SIT  0.33 µg/mL ERT | 0.2-0.3  SIT  0.3-0.5  ERT | 99.7% SIT  100.7%  ERT | Bulk &pharm. Forms | 0.9999  SIT  0.9997  ERT | Apr 2021 | [5] |
| RP-HPLC  Detection at  254nm | SP/MF | 200–1000 ng/mL  For both  SP&MF | 0.21µg/ml  SP  0.23µg/ml  MF | 0.64µg/mL  SP  0.71 µg/mL  MF | Inter- Day  0.19% SP  0.66%  MF | 99.6972  100.19 SP  99.456  100.09%  MF | Bulk&  Tablets  Form | 0.999  SP  0.998  MF | 2018 | [6] |
| CZE | SP/MH | 10-100  µg/mL SP  50:500 µg/mL MH | 0.49 µg/mL  SP  2.11 µg/mL MH | 1.48 µg/mL  SP  6.39µg/mL  MH | ≤1.50% (n = 3). | --- | Ph. prep & plasma | 0.9999  For both. | 2012 | [7] |
| Electrochemical sensor - | Sitagliptin | 1.7$\times$10^-8^-  2.2$\times$10^-5^  M | 9.1$\times$10^-9^ M | --- | --- | --- | Real sample | 0.9992 | Aug  2022 | [8] |
| DPV | sitagliptin | 100-2000 pM | 0.05 pM  nanoMIP1  0.06 pM  nanoMIP2  spiked human plasma | 0.18 pM  nanoMIP1  0.20 pM  nanoMIP2  spiked human plasma | 4.7%  NMIP1  4.3%  NMIP2 | --- | Plasma | 0.998  NMIP1  0.996  NMIP2 | May 2021 | [9] |
| MIP potentimetric sensors | sitagliptin | 5$\times$10^-6^-  10^-2^ M/L  MIP/MAA 2.5$\times$10^-6^:  10^-2^ M/L  MIP/2-VP | 2.6$\times$10^-6^ M/L  MIP/MAA 5.3$\times$10^-6^  MIP/2-VP | --- | >1% | 99.4 MIP/  MAA  99.7 MIP/2-VP | Bio-fluids | 0.9994  MIP/  MAA  0.999  MIP/2-VP | Aug 2014 | [10] |

**Table S2.**  Representation of different oxidation current and potential for different modified surfaces.

| **Electrode** | **I_oxidation_ (mA)** | **E_oxidation_(mV)** |
| --- | --- | --- |
| GCE | NA | NA |
| GCE/GO | 6 | -0.12 |
| GCE/NiO/CuO NPs | 14 | -0.13 |
| GCE/GO/ NiO/CuO NPs | 20 | -0.1 |

| 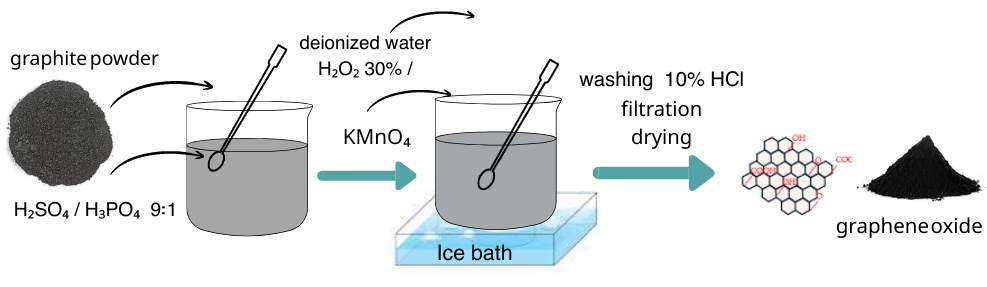 |
| --- |

**Figure S1.** Preparation of Graphene oxide using Hummers' method.

| 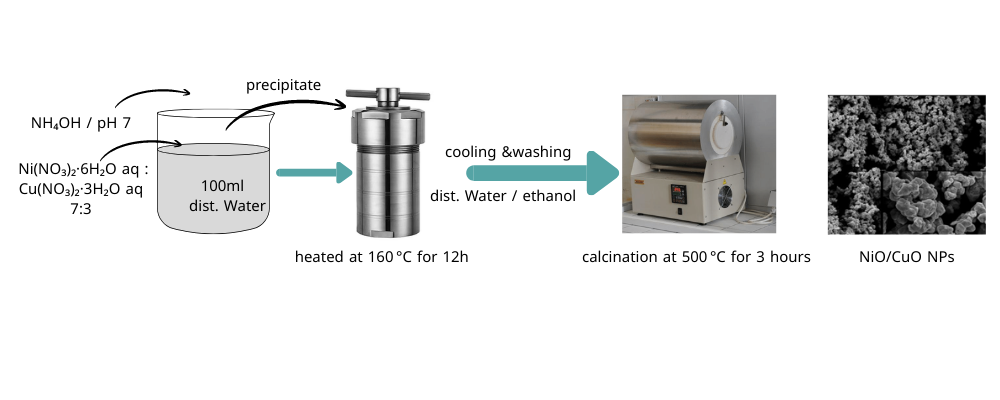 |
| --- |

**Figure S2.** Representation of preparation of NiO/CuO NPs by hydrothermal method.

| **Peak current Ip (µA)** | **SP conc (mM)** | **Mean (xˉ)** | **standard deviation (s)** | **RSD (%)** |
| --- | --- | --- | --- | --- |
| 19.779 | 1 | 19.8158 | 0.250 | 1.3 % |
| 19.865 |  |  |  |  |
| 19.424 |  |  |  |  |
| 20.010 |  |  |  |  |
| 19.686 |  |  |  |  |
| 20.131 |  |  |  |  |
| 3.80 | 0.1 | 3.83 | 0.0555 | 1.4 % |
| 3.85 |  |  |  |  |
| 3.79 |  |  |  |  |
| 3.75 |  |  |  |  |
| 3.92 |  |  |  |  |
| 3.86 |  |  |  |  |

**Table S3.** shows repeatability (intra-day precision n=6) calculated for 0.1 mM and 1 mM solutions of SP powder form by electrochemical method with the same modified electrode.

**Table S4.** shows reproducibility (inter-day precision n=3) calculated for (1, 0.1) mM solutions of SP powder form by electrochemical method with different modified electrodes.

| **Peak current Ip (µA)** | **SP conc (mM)** | **Mean (xˉ)** | **standard deviation (s)** | **RSD (%)** |
| --- | --- | --- | --- | --- |
| 19.779 | 1 | 19.809 | 0.167 | 0.8 % |
| 19.659 |  |  |  |  |
| 19.989 |  |  |  |  |
| 3.80 | 0.1 | 3.82 | 0.0464 | 1.2 % |
| 3.88 |  |  |  |  |
| 3.77 |  |  |  |  |

| 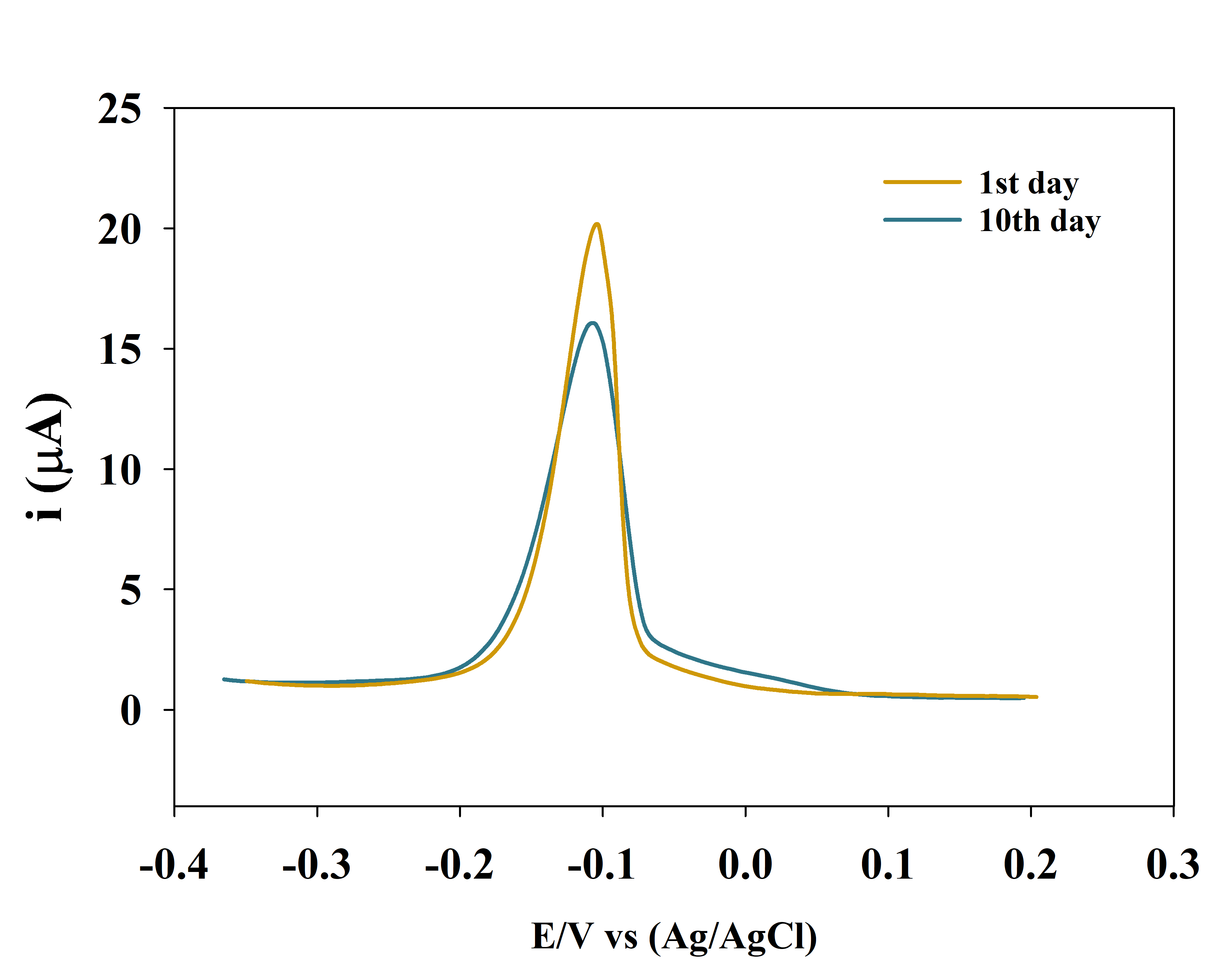 |
| --- |

**Figure S3**. DPV of inter-day stability for detection of SP drug in PBS.

**Sequence summary report for analysis of Sitagliptin phosphate mono hydrate (tablet) by HPLC analysis.**


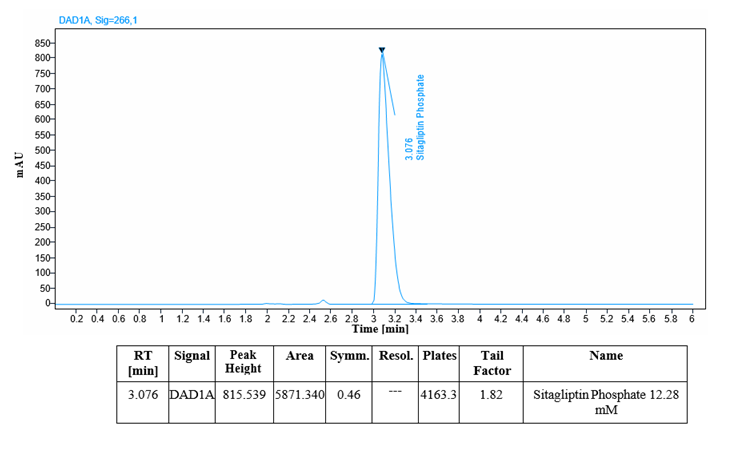


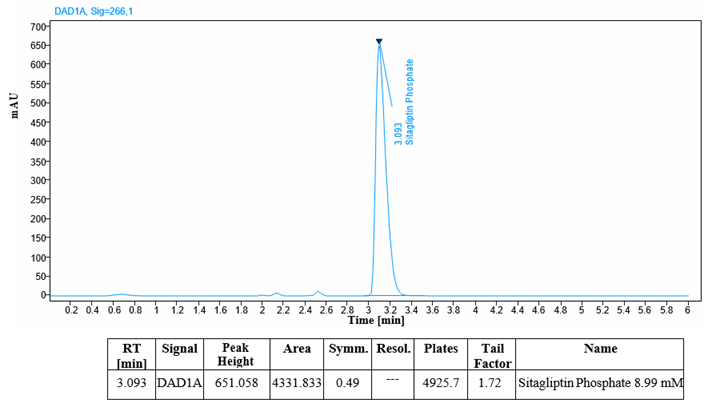


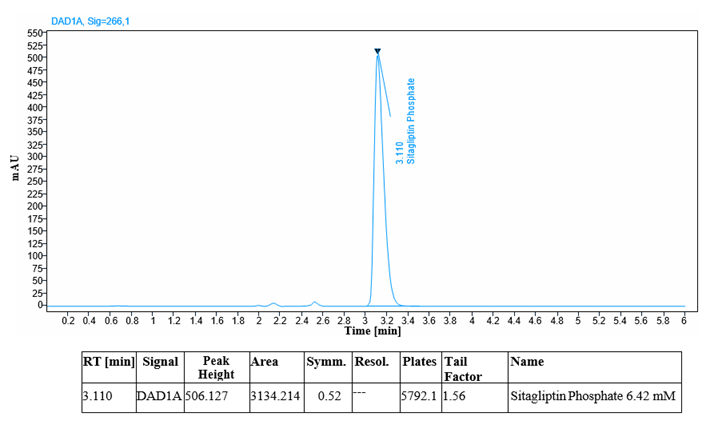


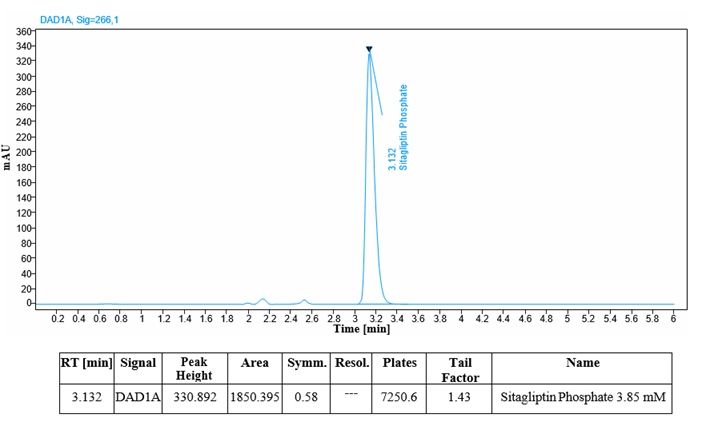


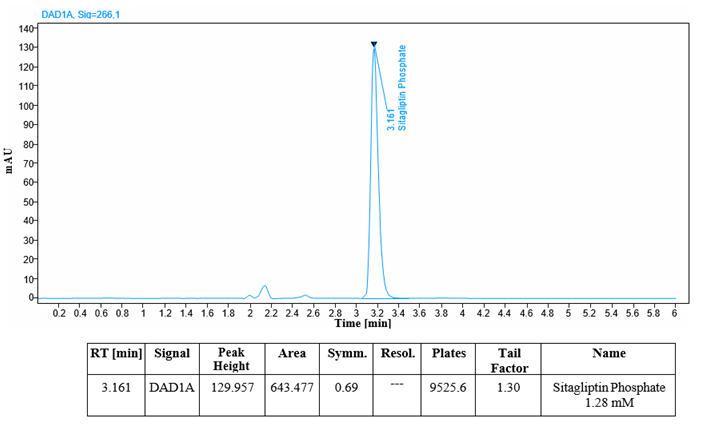


References

[1] J.Dr.Narendra. Govindasamy, Analytical Method Development and Validation of Sitagliptine Phosphate Monohydrate in Pure and Tablet Dosage Form by UV-Vis Spectroscopy., Research and Reviews: Journal of Pharmaceutical Analysis. 1 (2012) 19–23.

[2] A.& O.T.N.-A.& A.-J.Khalid. Mahmood, Colorimetric Determination Of Sitagliptin As An Oxidation Derivative Of Ninhydrin. , Systematic Reviews in Pharmacy 12 (2021) 175–178.

[3] M.N. Khan, M.N. Jan, Z. Ullah, Environmentally friendly protocol for the determination of sitagliptin phosphate in pharmaceutical preparations and biological fluids using l-tyrosine as a fluorescence probe, Luminescence (2023). https://doi.org/10.1002/bio.4567.

[4] M. Nashwahgadallah, Validated HPLC method for simultaneous determination of sitagliptin, metformine and atorvastatin in pure form and inpharmaceutical formulations, Int J Pharm Pharm Sci 6 (2014).

[5] R. k. , A.S. B, Novel stability-indicating RP-UPLC method for simultaneous estimation of sitagliptin and ertugliflozin in bulk and pharmaceutical formulations., Futur J Pharm Sci 7 (2021).

[6] S. Adsul, J.S. Bidkar, S. Harer, G.Y. Dama, RP- HPLC Method Development and Validation for Simultaneous Estimation for Metformin and Sitagliptin in Bulk and Tablet Formulation, Int J Chemtech Res 11 (2018). https://doi.org/10.20902/ijctr.2018.111149.

[7] M. Salim, N. El-Enany, F. Belal, M. Walash, G. Patonay, Simultaneous determination of sitagliptin and metformin in pharmaceutical preparations by capillary zone electrophoresis and its application to human plasma analysis, Anal Chem Insights 7 (2012). https://doi.org/10.4137/ACI.S9940.

[8] A.A. &Shafaatian, Bita. Abdi, Ultrasensitive Chemically Modified Carbon Paste Sensor for Reliable and Selective Potentiometric Determination of Trace Amounts of Sitagliptin in Real Samples. , ChemistrySelect 7 (2022).

[9] A.K.C.J.D.M.S.P.E.M.A.H.T.P.S.G.-C.A. Haq I, Determination of sitagliptin in human plasma using a smart electrochemical sensor based on electroactive molecularly imprinted nanoparticles. , Nanoscale Adv. 3 (2021) 4276–4285.

[10] A. &Galal, Hoda. Kamel, MIP-Based Biomimetic Sensors for Static and Hydrodynamic Potentiometric Transduction of Sitagliptin in Biological Fluids. , Int J Electrochem Sci 9 (2014) 4361–4373.
